# Drought Response and Genetic Variation in Scots Pine Seedlings' Provenances: Insights From High‐Throughput Phenotyping for Climate‐Resilient Forestry

**DOI:** 10.1111/eva.70157

**Published:** 2025-10-07

**Authors:** Eva Neuwirthová, Jan Stejskal, Zuzana Lhotáková, Jiří Korecký, Jaroslav Čepl, Antonín Nikodem, Klára Panzarová, Jana Albrechtová, Milan Lstibůrek

**Affiliations:** ^1^ Faculty of Forestry and Wood Sciences, Department of Forest Genetics Czech University of Life Sciences (CULS) Prague Prague Czech Republic; ^2^ Faculty of Science, Department of Experimental Plant Biology Charles University (CUNI) Prague Czech Republic; ^3^ Faculty of Agrobiology, Food and Natural Resources, Department of Soil Science and Soil Protection Czech University of Life Sciences Prague Prague Czech Republic; ^4^ Photon Systems Instruments, (PSI, Spol. s. r. o.) Drásov Czech Republic

**Keywords:** fluorescence, intraspecific variability, linear mixed models, needle functional traits, pedigreed seedlings, photosystem II, *Pinus sylvestris L.*, primary photosynthesis, resilience, SNP array

## Abstract

Scots pine ( *Pinus sylvestris* L.) is characterized by considerable intraspecific adaptive variability in response to environmental stress factors due to its wide geographical range. Adaptability is key for forestry, promising resilience against upcoming Europe's climate‐driven droughts. We studied three provenances of pedigreed Scots pine seedlings from distinct upland and lowland habitats in the Czech Republic. A water deficit was induced in 2‐year‐old, potted seedlings in a greenhouse. Their physiological responses to drought were investigated at the beginning of growing season during the development of new shoots, and after subsequent summer rewatering. (1) We analyzed several physiological traits to assess their effectiveness in detecting treatment effects: steady‐state quantum yield of PSII (QY Lss), maximum quantum yield of PSII (QY max), steady‐state non‐photochemical quenching (NPQ Lss), needle chlorophyll fluorescence ratio (SFR_R), and needle temperature normalized to ambient temperature (∆T), using a high‐throughput phenotyping unit. The divergence in SFR_R, QY max, QY Lss, NPQ Lss, and ΔT suggests that drought stress significantly impacts photosynthetic efficiency and heat dissipation, with recovery occurring after rewatering. (2) We detected differences within and among provenances utilizing a single nucleotide polymorphism genotyping array and linear mixed models integrating estimated genomic relationships to investigate genetic variation in needle functional traits in time. Throughout the experiment, heritability (*h* ^*2*^ ) varied widely among traits—with QY max and QY Lss showing the greatest variability (from 0 to 0.37), NPQ Lss exhibiting a narrower range aside from two outlier peaks, and SFR_R and ∆T displaying lower variability and lower *h* ^*2*^ values (0–0.24). The photosynthesis‐related traits (QY max, QY Lss) showed the highest genetic variation, underscoring their potential for early‐age phenotyping and selection of drought‐tolerant genotypes. These findings address practical problems in forest management, particularly in light of changing weather patterns and climate variability, and provide a foundation for advanced optically based, early‐age phenotyping to enhance forest resilience.

**Abbreviations**
∆T: temperature difference (between ambient air and needle temperature)
DAT: days after transplantation
DQC: dish quality control (used in genotyping)
EWT: equivalent water thickness
FR: red fluorescence
FRF: far‐red fluorescence
LMA: leaf mass per area
NPQ Lss: steady‐state non‐photochemical quenching
PSII: photosystem II
PWP: permanent wilting point
QY Lss: steady‐state quantum yield of photosystem II (PSII)
QY max: maximum quantum yield of photosystem II (PSII)
RGB: red, green, blue (imaging channels)
SFR_R: simple fluorescence ratio for chlorophyll content estimation
SNP: single nucleotide polymorphism
SRWC: soil relative water content

## 1 Introduction

Projected climate change is expected to lead to the degradation of a significant portion of the world's forests (IPCC 2022). Since the 1980s, the increasingly frequent coincidence of heat waves and drought has led to forest dieback (Allen et al. 2010; Breshears et al. 2021; IPCC 2022; Senf et al. 2018), fires, and disturbances (Rodrigues et al. 2023). Concurrently, tree mortality is associated with a broad‐scale decline in both timber yield and ecosystem services within European forests (Bose et al. 2020; Lindner et al. 2010).

The viability of locally adapted Scots pine populations under the anticipated future extreme conditions with prevailing long‐term drought and high‐temperature spells will depend on the species adaptive capabilities expressed by high phenotypic plasticity of functional traits in provenances to current global change conditions (Taeger et al. 2015). Due to biotic and abiotic stress factors acting on trees, shifts in forest species composition occur (Buras and Menzel 2019) resulting in changes in forest stability and productivity (García‐Valdés et al. 2020). The combined impact of drought and heatwaves creates pressure on the plants, and only those species or populations possessing a robust adaptive capacity in effectively moderating evaporation processes may have a chance to strive in future conditions (Rehschuh et al. 2022).

Scots pine ( *Pinus sylvestris* , L.) a pioneer tree species with a wide distribution, is highly resilient, and thanks to its general drought‐avoidance strategy combined with provenance‐specific physiological acclimations, it tolerates an extensive range of climatic conditions (Martínez‐Sancho et al. 2017; Seidel and Menzel 2016). Despite demonstrated resilience under adverse conditions, species distribution models suggest that its natural habitat may experience a reduction due to the impacts of climate change (Taeger et al. 2015). A recent study by Bose et al. (2020) showed that the response of mature lodgepole pine trees to drought varies significantly with tree growth history and drought exposure and concluded that physiological resilience to extreme drought may be limited by their pre‐drought growth, with more frequent and prolonged periods of drought overtaxing their acclimation capacity. In contrary southern drought‐adapted populations performed better in induced drought experiments (Seidel et al. 2019; Semerci et al. 2017). It implies that there is a variability in drought stress response in Scots pine emphasizing the importance of intraspecific variation of *P. sylvestris* provenances determined by different genetic backgrounds, which have to be taken into account before the deployment of considered provenances in assisted migration, for example (Taeger et al. 2015).

In light of these findings, it is clear that plant functional traits, with a special emphasis on those of leaves and roots, are instrumental in forecasting how ecosystems will react to environmental changes (Violle et al. 2007). Many leaf functional traits, including leaf biophysics, structure, and physiology, are detectable using optical methods such as remote sensing and high‐throughput phenotyping, can be combined with genomics (Santini et al. 2021). Intraspecific variation has emerged as a fundamental component of functional trait differences (Violle et al. 2007), as observed in the functional leaf traits of Scots pine studied along an elevation gradient (Carvalho et al. 2020). Intraspecific variation enhances our understanding of diversity across broad environmental gradients, as demonstrated in the case of subtropical *Pinus taiwanensis* Hayata (O'Sullivan et al. 2022). In the recent study, we found that hyperspectral phenotyping can differentiate phenotypic and genetic variations in Scots pine seedlings across different geographical locations, using locally adapted provenances (Stejskal et al. 2023).

Pine genus is considered having conservative stomatal behavior (Brodribb and McAdam 2013; Leo et al. 2014), characterized by early drought response in the form of stomatal closure (Ludovisi et al. 2017; Martínez‐Sancho et al. 2017) and reduction of the transpiration and photosynthesis rate (Seiler and Cazell 1990; Semerci et al. 2017). Moreover, regulation of stomatal aperture plays a vital role in maintaining plant temperature by promoting water loss and dissipating latent heat to prevent overheating (Rennenberg et al. 2006). The ecohydrological strategies used by Scots pine include isohydric behavior, which is characterized by efficient osmotic and stomatal regulation (Irvine et al. 1998), along with shallow root extension, accelerated metabolism, and precocious growth. These strategic physiological and morphological adaptations serve as key elements in preventing hydraulic failure, thereby increasing the resilience of Scots pine to drought and promoting climate change adaptability as reviewed by Dang et al. (2022). Across the Scots pine populations, a preference for mitigating the risk of hydraulic failure through priority regulation of stomatal control was shown, rather than by optimizing hydraulic traits such as maximum xylem and leaf maximum hydraulic conductivity and leaf‐to‐xylem area ratio (Martínez‐Sancho et al. 2017). The last‐resort hydraulic safety mechanism in Scots pine seedlings is leaf shedding (considered as the morphological response to drought stress; Nadal‐Sala et al. 2021).

In consequence to drought‐induced assimilation reduction, the performance of primary photosynthetic processes is expected to be affected. Several chlorophyll fluorescence kinetic parameters can be used as nondestructive indicators of tree drought response. The practical applications of chlorophyll fluorescence in forestry have been extensively described (Mohammed et al. 1995), encompassing the detection of various stress effects including the impact of among other things, also drought stress on phenology and ontogeny. In pine seedlings, chlorophyll fluorescence serves as a key indicator for detecting drought (Manes et al. 2001; Michelozzi et al. 2011), waterlogging stress (Pearson et al. 2013) and cold adaptation (Sofronova et al. 2016). It was also applied to assess changes in pigment composition in *P. sylvestris* needles under conditions associated with impending climate change (Wang et al. 2003). In drought‐stressed conditions, 6‐month‐old *P. halepensis* Mill seedlings (Michelozzi et al. 2011) and 2‐year‐old *P. sylvestris* seedlings exhibited a reduction in photosystem II (PSII) maximal photochemical efficiency, together with decreased root and shoot growth (Pearson et al. 2013). The dark‐adapted chlorophyll fluorescence indices in Scots pine needles reveal significant genetic variation (Čepl et al. 2016). At the needle level, chlorophyll content positively correlated with the Scots pine drought acclimation and its survival (Semerci et al. 2017) and the efficiency of PSII exhibited compensatory increase after the drought stress release (Seidel et al. 2019). Acclimation of Scots pine to drought reduces growth and increases root biomass allocation relative to leaves due to changes in gas exchange and photochemistry (Seidel and Menzel 2016; Taeger et al. 2015).

Drought stress tolerance is considered one of the pivotal traits targeted in forest tree breeding (Matallana‐Ramirez et al. 2021) in support of the concept of assisted migration. Enhancing trees' capacity to recover from water stress is essential for strengthening their long‐term resilience to changing moisture conditions and is thus a key objective in tree breeding programs (Zlobin 2024). Knowledge about the genetic basis of the drought stress response in gymnosperm remains limited (Ismael et al. 2022; Li et al. 2021; Moran et al. 2017), mainly due to the large and complex genome of conifers (Ahuja and Neale 2005; De La Torre et al. 2014). Drought resilience could be regarded as a complex polygenic trait, which can be studied by exploring associations with single nucleotide polymorphisms (SNPs). In the genus *Pinus*, drought resilience has been studied by SNP associations in *P. taeda* (Li et al. 2021; Lu et al. 2019, Lu et al. 2017), in *P. radiata* (Ismael et al. 2022) and in *P. halapensis* (Santini et al. 2021). Genetic studies on *Pinus sylvestris* have traditionally employed various nuclear and organellar markers (García‐Gil et al. 2003; Pyhäjärvi et al. 2008; Wachowiak et al. 2011). These advancements include methods that non‐specifically reduce the genome size for analyses, such as targeted sequencing (Tyrmi et al. 2020), reduced representation sequencing (Elshire et al. 2011; Hall et al. 2021), and RNA‐based sequencing (Ojeda et al. 2019). The integration of molecular markers into phenotypic prediction models improves accuracy and reduces bias, with Genomic Best Linear Unbiased Prediction (GBLUP) enhancing predictive ability for Eucalyptus growth traits (Cappa et al. 2019). Replacing pedigree‐based matrices with genomic relationship matrices refines genetic variance estimates including heritability, accelerates breeding cycles, and increases genetic gains and adaptability (El‐Kassaby et al. 2024). Narrow‐sense heritability estimates the proportion of phenotypic variance attributable to additive genetic effects and thus serves as a precursor to adaptive evolutionary change and selection response, as expressed in the breeder's equation, where the response to selection is proportional to the product of heritability and the selection differential (Isik et al. 2017).

The newly developed 50 K SNP genotyping array for Scots pine (Kastally et al. 2022) serves as an advanced tool with a wide range of applications in tree breeding activities. Genome‐wide association studies in Scots pine were conducted in connection to various wood (Traversari et al. 2022) and physiological traits, for example, drought tolerance (Baldi and La Porta 2022). Recently, a population genetic study using this SNP array described in detail the extent of fine‐scale spatial genetic structure and average dispersal distance in a large population of Scots pine from a continuous part of the distribution (Niskanen et al. 2024). However, only 0.4% of variation was explained by sampling location; thus, strong enough evidence of population‐level differences was not detected. The authors state that estimates of dispersal distance are relevant for practical applications, predicting responses to environmental changes, and understanding the balance between gene flow and other evolutionary factors, especially selection.

Facing the climate change‐induced challenges to forestry, there are calls for new technologies to improve understanding of tree drought responses and enhance forest resilience to drought and heat stress (Groover et al. 2025). In this study, we address this call by utilizing high‐throughput image‐based phenotyping in a controlled greenhouse experiment to non‐invasively determine the development and performance of plants under drought conditions over time in combination with genomic data from an SNP genotyping array. We monitored the offspring of three locally adapted Scots pine provenances from the Czech Republic with expected variation in needle functional traits induced by elevational adaptations, representing montane and lowland ecotypes.

The primary objective of this study was to investigate how key optically derived functional traits—specifically, primary photosynthetic activity, photoprotective responses, transpiration‐driven cooling, and chlorophyll content—respond to early‐season water limitation and subsequent summer rewatering in Scots pine seedlings. Additionally, we examined how these physiological traits reflect local adaptation across three provenances distributed along an elevation gradient and assessed their narrow‐sense heritability (*h* ^*2*^) to evaluate the genetic basis of trait variation at the provenance level. Genomic data obtained from the SNP chip array were used to reconstruct the genomic relationship matrix, serving as a refinement parameter in our linear models. Ultimately, this integrated approach aimed to elucidate the genetic and phenotypic bases of drought response, providing critical insights for developing more resilient reforestation materials.

## 2 Materials and Methods

### 2.1 Experimental Material and Design

Seed material represented the progeny of the parent trees originating from the three seed orchards from the Czech Republic: Plasy (P; 385 m. a. s. l), Trebon (T; 430 m. a. s. l.; lowland ecotype), and Decin (D; 465 m. a. s. l.; montane ecotype). Two‐year‐old Scots pine seedlings of the two ecotypes (lowland and montane) representing three locally adapted provenances with distinct ecological and geographical origins (Tables S1, S2 and Figure S1) were cultivated for 166 days (from February 21 to August 3, 2022) in semicontrolled greenhouse conditions (Figure S2). A total of 810 individual seedlings originated from 132 parent trees, whereby 791 individuals (303 from orchard Plasy, 299 from Trebon and 189 from Decin provenance), were used for the experiment. All juvenile plants were cultivated in Arboretum Sofronka (331 m. a. s. l) (Stejskal et al. 2023) for 2 years.

Seedlings were then transplanted into the 270 5‐l pots using a precisely given randomization scheme of three individuals per pot, every individual representing one of the three locally adapted provenances and parent trees. The seedlings were then transported to Drásov, subjected to a controlled irrigation and drought treatment scheme, including a rewatering phase. For 84 days, drought treatment was applied to 405 seedlings within 135 pots followed by rewatering and 36‐day long period of recovery.

**FIGURE 1:**
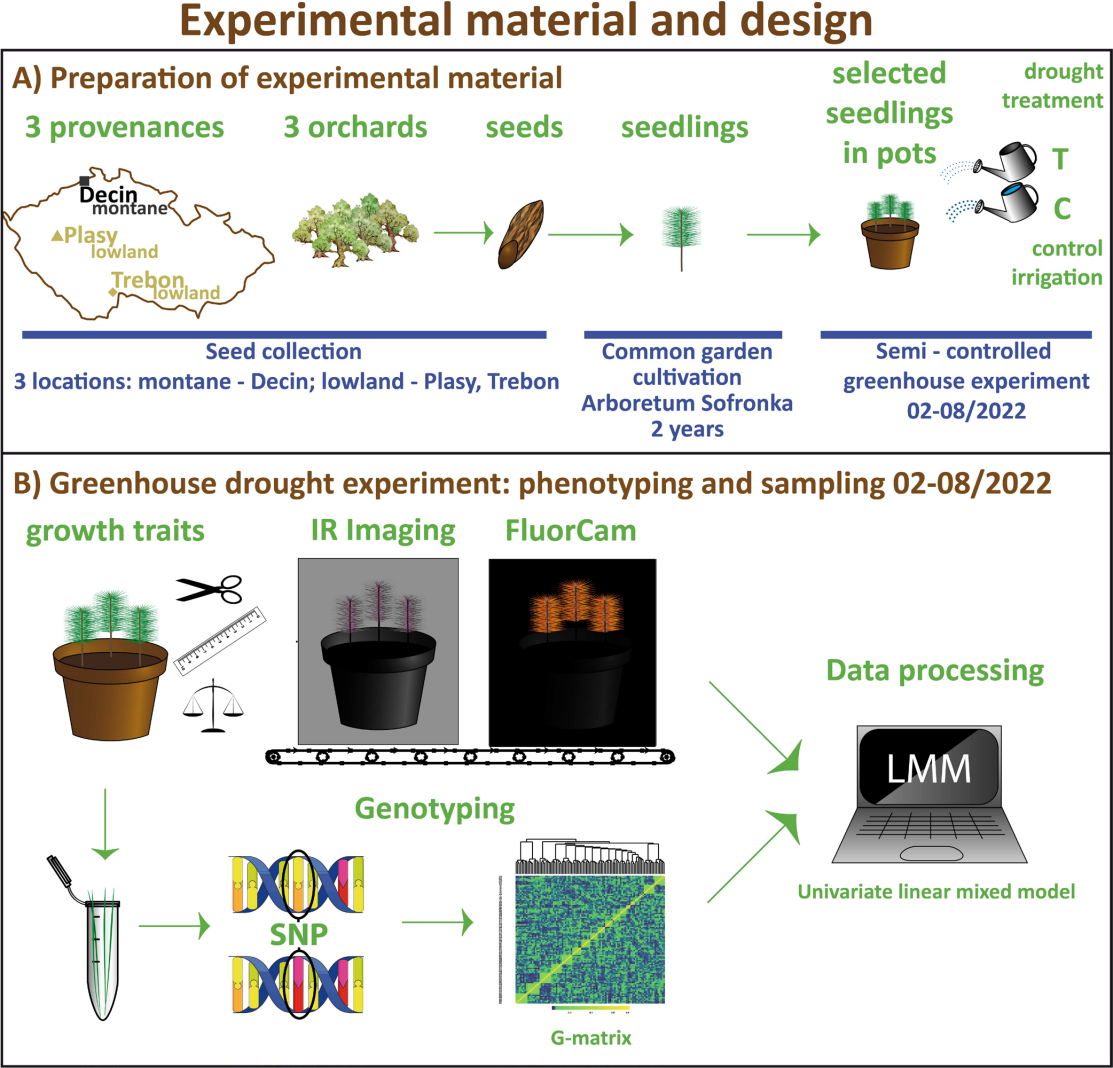
Experimental material and design. Summary of the preparation of experimental material and experimental design including setup, phenotypic and genotypic assessments, and statistical data analysis used in the study. (A) Preparation of experimental material: Seeds were collected from three locally adapted Scots pine provenances from three seed orchards (Plasy, Trebon, and Decin) representing two ecotypes (lowland—Plasy and Trebon; montane—Decin) in the Czech Republic. A total of 1778 seeds from 132 families were cultivated in a common garden at the Arboretum Sofronka for 2 years, yielding 810 seedlings. These seedlings were transplanted into 270 pots, with each pot containing three seedlings from different provenances and cultivated in semi‐controlled greenhouse conditions in phenotyping facilities (PSI Research Center, Drásov, Czech Republic) equipped with a phenotyping unit between February 21 and August 5, 2022. The seedlings were subjected to two different watering treatments: a drought treatment (T) and a control watering regime (C). (B) Greenhouse drought experiment. Growth traits such as needle length, root collar diameter, leaf mass per area, and needle water content were measured. High‐throughput phenotyping was performed using infrared (IR) imaging and chlorophyll fluorescence imaging (FluorCam) to assess plant physiological responses. DNA was extracted from 810 seedlings and genotyped using an SNP array generating a dataset of 32,998 SNPs. The genetic data were used to construct a genomic relationship matrix (G‐matrix). Data processing was performed using a univariate linear mixed model (LMM) to analyze phenotypic and genotypic associations.

Half of the randomly selected pots were subjected to drought treatment, while the remaining pots served as controls, receiving constant irrigation throughout the experiment. Each pot contained a seedling from a different orchard. Pots were filled with 4.1 kg of Profi‐substrat (Gramoflor GmbH & Co. KG, Campemoor 2, Germany) mixed with river sand (1:1) and for 1‐week plants were acclimated in semi‐controlled greenhouse conditions. The preparation of experimental material and overall experimental design is described in Figure 1.

In our 164‐day experiment in a semi‐controlled greenhouse condition, we monitored environmental variables using five MultiSensors (PSI, Drásov, Czech Republic) equally distributed over the inner cultivation area for continuous temperature, photosynthetic active radiation (PAR), and air humidity monitoring (Figure S2). In addition, outer environmental sensor was used to record external weather conditions. Our findings highlight the dynamic interplay of these factors and reveal seasonal trends and occasional fluctuations driven by ambient environmental effects.

### 2.2 High‐Throughput Phenotyping Setup and Soil Moisture Control

#### 2.2.1 Automated Phenotyping Platform Setup

The PlantScreen Modular System (PSI, Drásov, Czech Republic) is a fully automated phenotyping platform that encompasses an imaging area including an acclimation tunnel ensuring standardized light conditions prior to entering the imaging units and greenhouse‐located cultivation area (Findurová et al. 2023). Imaging sensors implemented for the digital analysis are a kinetic chlorophyll fluorescence imaging unit, a thermal imaging unit, a top and a multiple angle side view RGB imaging unit, and VNIR and SWIR hyperspectral imaging unit. Furthermore, a weighing and watering station is integrated for controlled water and nutrient delivery on a single pot level. Each pot was daily automatically moved on a conveyor belt between the cultivation greenhouse‐located area and the imaging area with the automatic laser height measuring unit, light/dark acclimation unit, robotic‐assisted imaging units, and the weighing and watering unit to maintain defined soil relative water content (SRWC) in each pot. All raw digital data were automatically processed through the PlantScreen Analyzer software (PSI, Drásov, Czech Republic).

In this study, we present data from the chlorophyll fluorescence imaging unit and a thermal imaging unit. Measurements were performed from March 14 to August 2, 2022, encompassing 43 measured time points for light‐adapted chlorophyll fluorescence, 37 instances for dark‐adapted chlorophyll fluorescence, 24 instances for multicolor fluorescence for chlorophyll content estimation (Lichtenthaler 2021), and 42 instances for infrared thermal camera assessments. Phenotypic assessments for light‐adapted fluorescence, chlorophyll content estimation, and infrared imaging were conducted between 9:00 and 18:00 CET, while assessments for dark‐adapted chlorophyll fluorescence occurred between 22:00 and 4:00 CET.

On a daily basis, the position of the pots containing tree seedlings was automatically randomized to minimize spatial effects and diurnal cycle‐induced differences between treatments (watered and drought‐treated plants) and among genotypes.

#### 2.2.2 Soil Moisture Methodology Design: Irrigation, SRWC, and PWP Assessment

The irrigation protocol controlling the water content of individual pots was operated by an automatic watering and weighing unit of the PlantScreen Modular System. During the plant acclimation ongoing after the first week after transplantation, all pots were watered up to soil relative water content 70% field capacity (SRWC) upon transplantation. The SRWC (%) was determined based on the comparison between the soil fresh weight (FW), soil saturated weight (SW), and soil dry weight (DW): (FW−DW)/(SW−DW) × 100. The pots containing the control group of seedlings were irrigated to a reference weight where the soil relative water content (SRWC) was maintained at 50% throughout the duration of the experiment (166 days). For the drought‐treated group, the irrigation protocol was divided into multiple phases. From the first day after transplantation (DAT 1) (February 22nd) up to DAT 43 (April 6th) watering was maintained on 50% SRWC as the control group. A gradual reduction in watering of the drought‐treated group started from April 7th (DAT 44), when SRWC was reduced to 20%; then to 18% from May 16th (DAT 83) and to 15% from June 1st (DAT 99). The reduction to 10% SRWC started on June 17th (DAT 115) and continued until irrigation ceased on June 24th (DAT 122), when SRWC reached 0%. After 5 days of 0% SRWC (DAT 122–127), stepwise rewatering to 50% SRWC of the treated group began on June 30th (DAT 128), and 50% SRWC was maintained until the end of the experiment on August 3rd (DAT 162).

The soil water content corresponding to the permanent wilting point (PWP, at *h* = 15,000 cm) was obtained on the 100‐cm^3^ undisturbed soil samples (three replicates for each scenario) using a pressure plate apparatus (Dane and Topp 2020). In our study, PWP was calculated as 20% of SRWC, based on measurements from May 16th (DAT 83).

### 2.3 Functional Traits Measured and Measurement Frequency

Throughout our experiment, we examined the variation of functional traits: growth‐related functional traits were measured once during the experiment, while optically detected functional traits were measured repeatedly using automatized phenotypic measurements during the whole period of the experiment.

Manual measurements of growth‐related functional traits were made for each seedling in situ: needle length (NL, cm) and root collar diameter (RCD, cm) were measured on DAT 135 (July 7th—7 days after rewatering) and leaf mass per area (LMA mg·cm^−2^), equivalent water thickness (EWT mg·cm^−2^) collected at DAT 168 (August 9th—40 days after rewatering) (see Data Availability).

Additionally, response of Scots Pine seedlings to early‐season drought and summer rewatering was optically assessed using a phenotyping platform for five needle functional traits: light‐adapted steady‐state PSII quantum yield (QY Lss) and maximum quantum yield of PSII (QY max). QY Lss and QY max indicate the current state and maximum efficiency of PSII, providing valuable insights into the photosynthetic performance of the seedlings under varying environmental conditions. Steady‐state non‐photochemical quenching (NPQ Lss) reports on the impossibility of light energy use for photochemical electron transfer. Additionally, we estimated needle chlorophyll content through simple chlorophyll fluorescence ratio (SFR_R) calculation and measured the temperature difference (∆T°C) between the ambient air temperature and the needle, corresponding to effectiveness of transpiration cooling.

Functional leaf traits such as QY Lss, QY max, NPQ Lss, and ∆T were measured twice a week, except for the SFR_R index, which was measured once a week. These measurements were conducted using a fully automated phenotypic system, with at least 40 measurements for each parameter and at least 20 measurements for the SFR_R index throughout the season, see the Data Availability section. Detailed description of measuring growth functional leaf traits is given in Table 1.

**TABLE 1:** Summary of measured traits, their abbreviations, and measurement details: For each trait, the table lists its abbreviation, a brief description, the calculation method, sensor or measurement method used, measurement frequency, and total number of measurements during the experimental period. Growth‐related traits (needle length, root collar diameter, leaf mass per area, and needle water content) were measured manually at the end of the experiment. Optical traits related to photosynthetic performance—such as light‐adapted and dark‐adapted chlorophyll fluorescence parameters (QY Lss, QY max, NPQ Lss), the simple fluorescence ratio (SFR_R), and the needle temperature difference (ΔT)—were obtained using the PlantScreen Modular System with its integrated imaging units.

Trait (Abbrev.)	Description	Calculation	Sensor/Method	Measurement frequency	No of meas.
NL	Needle length	Measured length (cm)	Manual measurement (digital caliper)	Once in the experiment	1
RCD	Root collar diameter	Measured length (cm)	Manual measurement (digital caliper)	Once in the experiment	1
LMA	Leaf Mass per Area	LMA = Dry Weight/Leaf Area	Manual: fresh/dry weight, leaf area scanning (EPSON V600 + ImageJ)	Once in the experiment	1
EWT	Equivalent water thickness	Ewt = (fresh weight−dry weight)/needle area	Manual: fresh/dry weight (oven‐dried 72 h/60°C)	Once in the experiment	1
SFR_R	Simple Fluorescence Ratio for chlorophyll content estimation	SFR_R = 735 nm/685 nm	Multi‐color fluorescence imaging in PlantScreen Modular System (FluorCam FC1300/8080‐15)	Once per week (~7 days)	25
QY Lss	Steady‐State Quantum Yield of PSII	QY Lss = Fm Lss−Ft/Fm Lss	Light‐adapted chlorophyll fluorescence (FluorCam FC1300/8080‐15)	Twice a week (~3 days)	44
QY max	Maximum Quantum Yield of PSII	QY max = Fm−Fo/Fm	Dark‐adapted chlorophyll fluorescence (FluorCam FC1300/8080‐15)	Twice a week (~3 days)	38
NPQ Lss	Steady‐State Non‐Photochemical Quenching	NPQ Lss = Fm−Fm Lss/fm Lss	Dark‐adapted quenching kinetics (FluorCam FC1300/8080‐15)	Twice a week (~3 days)	38
ΔT	Temperature Difference (needle vs. ambient air)	ΔT = Tneedle−Tair	Thermal imaging (InfraTec VarioCam HEAD 820) in PlantScreen Modular System	Twice a week (~3 days)	43

#### 2.3.1 Growth‐Related Functional Traits

For each individual seedling, four needles were collected and used for further analyses: RCD and NL were measured in situ using a digital caliper. For LMA and EWT, samples were collected within 1 day, and those fresh four needles were stored in the preweighed 2‐ml tubes and directly after the sampling were stored in the refrigerator. Fresh weights were determined along with the preweighed tubes, followed by the measurement of leaf area. The leaf area of fresh needles was scanned using an EPSON Perfection V600 Photo scanner with a top lamp, with a resolution of 800 dpi, and analyzed using ImageJ software. Fresh needles were dried in an oven at 60° for 72 h. Dry needle weights were determined for each individual seedling and used to calculate equivalent water thickness (EWT) using the formula: EWT = (FW−DW)/A; where FW is fresh weight, DW is dry weight, and A is needle projection area. Dry needle weight and needle projection area were used to calculate leaf mass per area (LMA) using the formula: LMA = DW/A.

#### 2.3.2 Optically Assessed Functional Traits Using a Phenotyping Platform

##### 2.3.2.1 Needle Temperature Assessment Using Thermal Imaging to Characterize Transpiration Cooling

The determination of needle temperature difference from the ambient air corresponds to the effectiveness of needle transpiration, wherein a lower value of the needle temperature signifies more efficient transpiration cooling. Thermal images were obtained using an InfraTec thermal camera (VarioCam HEAD 820 (800)) with a resolution of 1024 × 768 pixels, thermal sensitivity of < 20 mK, and thermal emissivity value set default to 0.95. The infrared imaging box contained a camera mounted on a side view oriented robotic arm for side view imaging, a rotating table with precise plant positioning, and automatically controlled heated wall on the opposite side to the thermal camera. PT1000 temperature sensors embedded in the wall were used for automatic feedback control of the heated wall temperature, maintaining it at an offset of 8°C above ambient to enhance contrast during image processing. The thermal images were acquired in darkness using line scan mode as described in Findurová et al. (2023) with a scanning speed of 30 Hz and each line consisting of 768 pixels. The imaged area was 350 × 1005 mm (height × width).

Thermal images were captured after 1 min light adaptation in the acclimation tunnel. The images were acquired in darkness from the side view. The imaging conditions, plant location, and camera settings were kept constant throughout the experiment. The canopy temperature of each plant was automatically extracted with PlantScreen Analyzer software by RGB plant mask application, subtraction of background, and pixel‐by‐pixel integration of values across the whole plant surface area. To minimize the effect of environmental variability and differences in the timing of image acquisition between plants, needle temperature was normalized to the ambient air temperature inside the imaging unit and calculated: ∆T = needle T−air T. Negative values of the ∆T mean that the leaf is cooler than the surrounding air and vice versa. Lower values of ∆T show more effective transpiration cooling.

##### 2.3.2.2 Fluorescence Needle Traits Measurement

To evaluate the impact of drought stress on the performance of primary photosynthesis in Scots Pine seedlings, chlorophyll fluorescence measurements from the top view were conducted utilizing an enhanced FluorCam FC1300/8080‐15—r‐o/white, six colors pulse‐amplitude modulated (PAM) imaging system, integrated into the PlantScreen Modular System. In the chlorophyll fluorescence imaging unit, a TOMI‐2 high‐resolution camera (resolution of 1360 × 1024 px, frame rate 20 fps and 16‐bit depth) with a 7‐position filter wheel is mounted on a robotic arm. The camera is positioned in the middle of the multicolor LED light panel with dimensions of 1326 × 1586 mm. The LED panel was equipped with 4 × 240 red‐orange (618 nm), 120 cool‐white LEDs (6500 K) and 240 far‐red LEDs (735 nm) distributed equally over the imaging area of 80 × 80 cm. Additional six colors lights (UV, 365 nm; royal blue, 450 nm; blue, 470 nm; green, 530 nm; amber, 600 nm; cyan, 505 nm) are integrated into the panel for static fluorescence measurements and multicolor fluorescence imaging. Three distinct protocols were implemented based on the traits under consideration:

#### 2.3.3 Steady‐State Quantum Yield of PSII (QY Lss)

Measurements of the current state of PSII were taken during daylight hours as light‐adapted measurement. Prior to measurement, plants were allowed to acclimatize for at least 2 h upon sunrise in the cultivation area under the cultivation growth light conditions (minimum 320 μmol m^−2^ s^−1^). This ensures that photosynthesis and stomatal regulation were in a steady state (Smith et al. 2004).

Prior chlorophyll fluorescence imaging, plants were transported through the acclimation tunnel equipped with cool‐white LEDs (6500 K), where they were adapted to the light for 5 min as described by Findurová et al. (2023). Cool‐white LED actinic light (1200 μmol m^−2^ s^−1^ PAR) was used for plant adaptation and chlorophyll fluorescence measurements. The measuring protocol was set as follows: plants were exposed to 3 s of cool‐white actinic light (500 μmol m^−2^ s^−1^) to determine steady‐state fluorescence in the light‐adapted steady state (Ft_Lss), followed by a 1000‐ms saturation pulse (4200 μmol m^−2^ s^−1^) to determine maximum fluorescence in the light‐adapted steady‐state (Fm_Lss). Afterward, the actinic light was turned off, far‐red light (735 nm) was turned on, and PSII was relaxed in the dark for 2 s to determine the minimum fluorescence in the light‐adapted steady state (Fo_Lss). The extracted parameter was the light‐adapted steady‐state (actual) quantum yield of PSII (QY Lss), calculated as (Fm_Lss—Ft)/Fm_Lss.

#### 2.3.4 Maximum Quantum Yield of PSII (QY Max) and Non‐Photochemical Quenching (NPQ Lss)

Dark‐adapted measurements were initiated at least 1 h after the dusk. Plants were further dark‐adapted for 5 min in the adaptation tunnel, followed by automatic transportation to the light‐isolated chlorophyll fluorescence imaging unit. A 5‐s flash of light was applied to record minimal fluorescence in the dark‐adapted state (Fo), followed by an 800‐ms saturation pulse (4200 μmol m^−2^ s^−1^) to determine the maximum fluorescence in the dark‐adapted state (Fm). The maximum quantum yield of dark‐adapted samples was calculated as QY max = (Fm − Fo)/Fm.

Plants were relaxed in the dark for 3 s and then subjected to 60 s of cool‐white actinic lights to drive photosynthesis and measure the peak rise in fluorescence (Fp). For the quenching kinetics protocol, additional saturation pulses were applied at 18, 38, and 58 s during actinic illumination, corresponding to L1, L2, and Lss states at a constant photon irradiance of 550 μmol m^−2^ s^−1^ to acquire the maximum fluorescence in the light‐adapted states (Fm´). The steady‐state fluorescence in the light‐adapted state (Ft) was acquired just before the saturation pulses. Afterward, the actinic light was turned off, far‐red light (735 nm) was turned on, and PSII was relaxed in the dark for 2 s to determine the minimum fluorescence in the light‐adapted steady state (Fo_Lss).

The PlantScreen Analyzer software was used for the automated fluorescence feature extraction by mask application, background subtraction, and parameter calculation based on the fluorescence levels of Fo, Fm, Fp, Ft, Fo_Lss, and Fm´, which were estimated by integrating pixel‐by‐pixel values across the canopy area. Steady‐state non‐photochemical quenching (NPQ Lss) of chlorophyll fluorescence was calculated as: NPQ Lss = Fm−Fm_Lss/Fm_Lss.

Dark‐adapted measurements for single time points were compiled from measurements acquired during two successive nights as only 135 holders could be measured during the phase of single night.

#### 2.3.5 Multicolor Fluorescence for Chlorophyll Content Estimation

Imaging the chlorophyll fluorescence ratio at red to far‐red wavelengths provides a relative measurement (i.e., index) of the chlorophyll content of leaves by generating fluorescence in leaf tissues using multiple light sources (Ghozlen et al. 2010). The chlorophyll content is positively correlated to the ratio of chlorophyll fluorescence measured in the far‐red to the chlorophyll fluorescence measured in the red part of visible radiation (Buschmann 2007; Lichtenthaler 2021; Lichtenthaler et al. 1986). The multicolor fluorescence module of FC1300/8080‐15—r‐o/white, six colors vs2 PAM chlorophyll fluorometer was used to measure the simple chlorophyll fluorescence ratio. Subsequent measurements were performed under the red‐orange light excitation (_R), and the ratio is expressed as follows: a simple chlorophyll fluorescence ratio (SFR_R), defined as the far‐red fluorescence emission (FRF_R, 735 nm) divided by the red fluorescence emission (RF_R, 685 nm) SFR_R = FRF_R/RF_R (Ghozlen et al. 2010). The values were extracted from PlantScreen Analyzer software and used as a noninvasive indicator of leaf chlorophyll content (Lichtenthaler 2021).

### 2.4 Genomic Data Filtration, Genetic Differentiation of Provenances, and Construction of the G Matrix

To generate SNP calls (Kastally et al. 2022) for 790 Scots pine genotypes, we used the Axiom Analysis Suite Software (Thermo Fisher Scientific). SNP data underwent quality control with filters set for a dish quality control (DQC) value of at least 0.82, a quality control (QC) call rate of 90% (resulting in 47,712 SNPs), Fisher's linear discriminant (FLD) score greater than 5 (33,489 SNPs), homozygous FLD (homFLD) score over 10 (33,381 SNPs), and a minor allele frequency (MAF) above 0.01 (31,677 SNPs), while default threshold QC parameters were preserved. We retained only SNPs classified as PolyHighResolution conversion type, narrowing down to a set of 31 611 high‐quality SNPs (see the Data Availability section).

Due to the relatedness of genotyped half‐sibling offspring, allele frequencies were expected to deviate from Hardy–Weinberg equilibrium (HWE). To address this, SNP selection was performed in two steps: First, SNPs in disequilibrium were identified within each parental provenance separately; then these were selected within the offspring provenance.

Additionally, we screened loci for heterozygosity, as excessive heterozygosity may indicate genotyping errors or paralog issues. Loci with > 60% heterozygotes were excluded (e.g., Suissa et al. 2024). While the selected SNPs no longer conformed to HWE in offspring, this approach was preferred over direct HWE filtering in the offspring provenance. We also excluded SNPs that significantly deviated from Hardy–Weinberg equilibrium (*p* < 0.001). The final set of SNPs common to all three provenances, totaling 20,216 SNPs (see Data Availability). The provenance genetic analysis was conducted using discriminant analysis of principal components (DAPC) with functions from the R package adegenet (Jombart and Ahmed 2011). To balance discrimination power and overfitting, we applied the optim.a.score() function to determine the optimal number of retained principal components (PCs), resulting in n.pca = 40. Genetic differentiation among provenances was assessed using Fst and Nei's D, as implemented in the StAMPP package. From the filtered SNP dataset, the genomic relationship matrix (G‐matrix) was constructed according to Yang et al. (2010), employing the R package AGHmatrix (Amadeu et al. 2016) (see Data Availability).

### 2.5 Analyses

For statistical analyses, the R software version 4.0.4 (R Core Team, 2021) and the ASReml library for R version 4 (Butler et al. 2018) were used. Although repeated‐measures models would have been the preferred analytical approach, convergence issues prevented their implementation (Table S9). Therefore, a separate univariate linear mixed model was fitted for each time point to evaluate needle functional traits, using the following form:

(1) $$y=1\mu +X_{1}\beta_{s}+X_{2}\beta_{t}+X_{3}\beta_{\mathrm{st}}+\;G\beta_{a}+X_{4}\beta_{p}+e$$

where 𝒚 corresponds to the phenotypic value; 𝜇 is the overall mean effect; $\beta_{s}$ is a fixed effect of the provenance (D, P, T); $\beta_{t}$ is a fixed effect of the treatment; $\beta_{\mathrm{st}}$ denotes the interaction effect between provenance and treatment; $\beta_{a}$ is the random additive genetic value, $\beta_{b}$ is the random effect of the pot. X are respective incidence matrices associated with fixed and random effects; G is the genomic relationship matrix, and 𝒆 is the random error term.

Wald test was implemented to evaluate the fixed effects of provenance, treatment, and their interaction on all measured traits (Butler et al. 2018). Respective provenance means for individual traits were estimated as best linear unbiased estimates (BLUEs). Ninety‐five percent confidence intervals, incorporating variance arising from residuals after accounting for random effects, were calculated using the asremlPlus package (Brien 2025). Significance of pairwise contrasts was assessed using SED‐based *p*‐values, reported both as unadjusted and as FDR‐adjusted values (Benjamini & Hochberg method) using the p.adjust function across all tests within a given DAT.

Individual, narrow‐sense heritability (*h* ^*2*^) was estimated as

(2) $$h^{2}=\frac{\sigma_{\alpha }^{2}}{\left(\sigma_{\alpha }^{2}+\sigma_{p}^{2}+\sigma_{e}^{2}\right)}$$

where $\sigma_{\alpha }^{2}$ is the additive genetic variance; $\sigma_{p}^{2}$ denotes the variance attributable to pot effects, and $\sigma_{e}^{2}$ is the residual variance. We derived the approximate standard errors of *h* ^*2*^ using the Delta method. The significant values of *h* ^*2*^ in the results are based on likelihood ratio tests (LRT; Isik et al. 2017). The LRT compares the goodness of fit between the null model (the model fitted without the genetic component, i.e., the $G\beta_{a}$ term) and the model based on Equation (1).

## 3 Results

### 3.1 Response of the Functional Traits to Drought, Related to Seedlings' Origin

Our analyses not only considered differences between control and treated plants but also explored variation among and within three locally adapted provenances of seedlings' origin: Decin (D), Plasy (P), Trebon (T) in studied functional traits (Table 1 and Tables S3–S8).

### 3.2 Response of Growth‐Related Functional Traits to Drought in Locally Adapted Provenances

Our analysis revealed significant differences in all growth‐related parameters between control and treated plants, as indicated in Table S3. During the advanced phase of the drought period, we noticed remarkable needle shedding. Just before the rewatering (DAT 127, June 30th), we detected needle shedding in 201 individuals.

The majority of seedlings with pronounced needle shedding belonged to the control group—in total 179 individuals (89% of all shedding seedlings). Of these, 137 were from lowland progenies (Plasy: 64; Trebon: 73), while only 42 individuals from the montane progeny (Decin) shed needles, highlighting that needle shedding was much more common in lowland than in montane progenies. In the drought‐treated group, only 22 seedlings shed needles. A similar pattern was observed among the lowland progenies (Plasy: 12; Trebon: 7), whereas only three seedlings from the montane progeny (Decin) shed needles, which is less than half the number observed in the lowland progenies.

**FIGURE 2:**
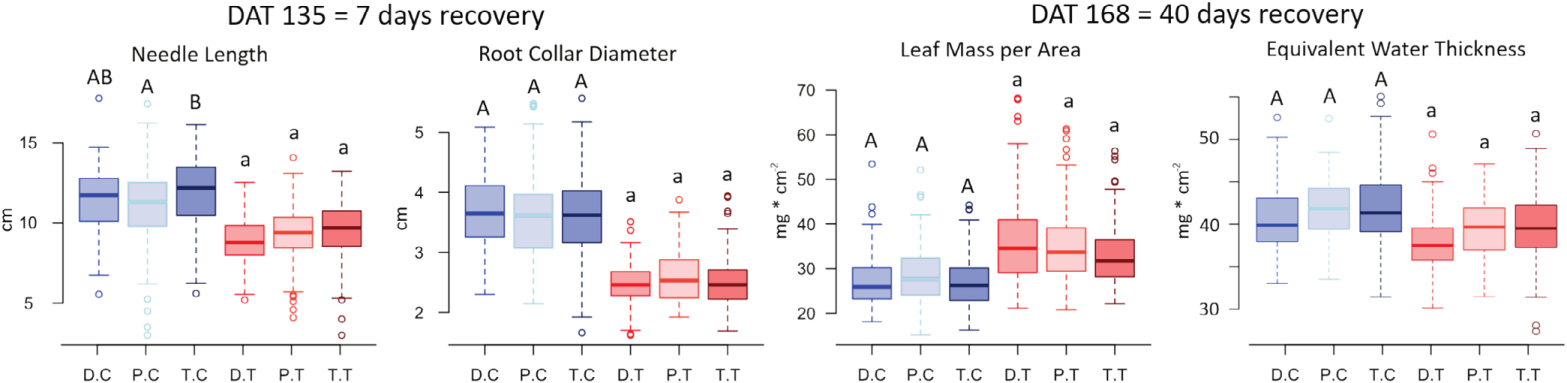
Measured growth traits: (Needle length and root collar diameter) sampled at 7 days (DAT = 135); and (leaf mass per area and equivalent water thickness) at 40 days (DAT = 168) of the recovery phase following the early‐season drought period. Control plants (D.C, P.C, T.C) are in blue, treated plants (D.T, T.T, P.T) in red. Different letters above boxes denote significant differences (α = 0.05) among provenances (D—Decin, P—Plasy, T—Trebon) in both control and treated groups separately).

In control plants, no significant differences were observed among provenances (Decin, Plasy, and Trebon) for any parameter except needle length (NL); here, the Trebon progeny exhibited significantly longer needles than the Plasy provenance, despite both being lowland ecotypes (Figure 2).

### 3.3 Response of Needle Functional Traits to Drought During the Season

**FIGURE 3:**
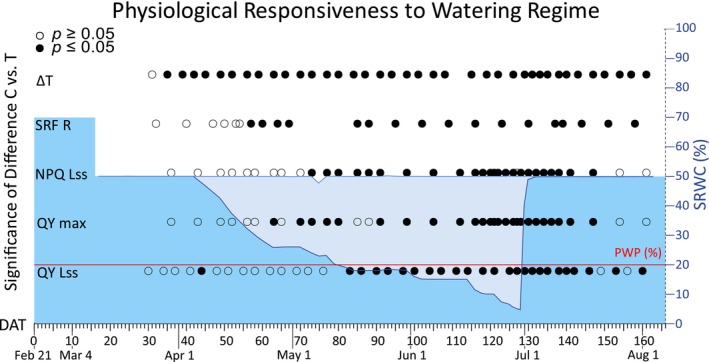
Measured soil relative water content (SRWC) and differences of the five key needle functional traits: (QY Lss, QY max, NPQ Lss, SFR_R, ∆T) control (C) and treated plants (T) evaluated by univariate linear mixed model, explicitly controlling for treatment, provenance and random factors (genetic relationships and pot effects). The left Y‐axis represents needle functional traits (QY Lss, QY max, NPQ Lss, SFR_R, ∆T). X‐axis: Days after transplantation (DAT 0–166). The impact of differential watering regimens on five key needle functional traits over 166 days (days after transplantations = DAT) from February 21, 2022 to August 7, 2022. ANOVA‐style Wald test of fixed factor treatment is represented by dots. Full dots denote significant (*p* ≤ 0.05) and open symbols non‐significant (*p* ≥ 0.05) differences between C and T plants for the functional traits. The red line signifies the maximum drought (when the soil moisture below the permanent wilting point (PWP)), determined at 20% of the soil relative water content—a critical amount of water available for the plants, serves as a crucial water stress threshold in the specific soil type employed in our study. Two plant groups were examined: C consistently watered at 50% SRWC, T subjected to progressive substrate dry‐down. The right vertical axis illustrates SRWC for C received a steady 50% watering level, while T irrigation levels are denoted by blue line. The blue region represents gravimetrically acquired SRWC for C (lighter blue) and T (darker blue) over 166 days (February 21, 2022 to August 7, 2022).

In Figure 3, we report the sensitivity of five needle optical‐based functional traits (QY Lss, QY max, NPQ Lss, SFR_R, ∆T) to drought treatment over the 166‐day experiment with respect to relative soil water content (SRWC). This initial visualization allowed us to determine the effect of irrigation regime on each functional trait.

**FIGURE 4:**
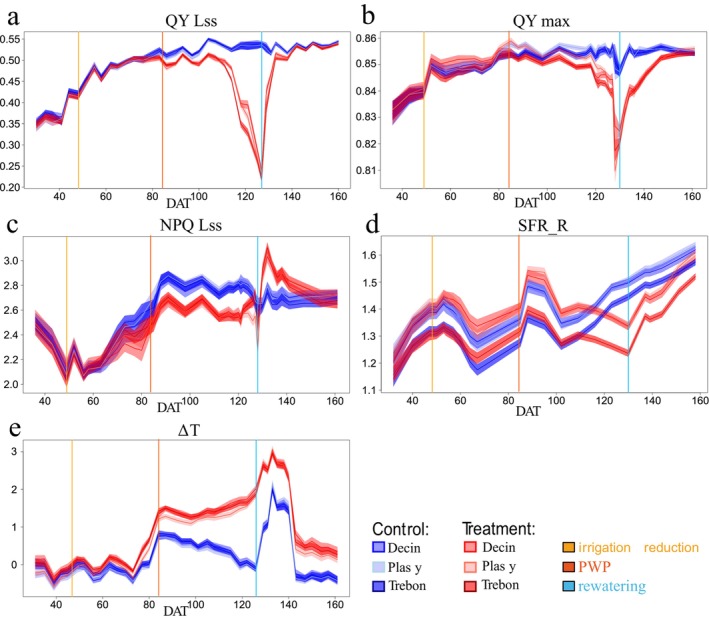
Mean trait values (*Y*‐axis) for five needle functional traits: (a = QY Lss, b = QY max, c = NPQ Lss, d = SFR_R, e = ∆T), obtained by univariate linear mixed model with 95% confidence interval, incorporating variance arising from residuals after accounting for random effects; note that overlaps between confidence intervals for individual means do not necessarily indicate non‐significance, as statistical significance is assessed directly through pairwise comparisons using residual variance alone; in X‐axis days after transplantation (DAT 0–166). Control plants from Decin (D), Plasy (P), and Trebon (T) are represent by blue; treated plants from D, T, and P are represent by red. The yellow line represents irrigation reduction at DAT 44, the orange line represents the permanent wilting point (PWP) from DAT 83, when water was not available for the plants in soil (SRWC under 20%), and the blue line represents rewatering at DAT 128.

The two chlorophyll fluorescence parameters—steady‐state and maximum quantum yield of PSII (QY Lss, QY max) evolved similarly throughout the experiment. During decreasing irrigation, both fluorescence yields of treated plants decreased due to the water stress. In the stressed group, QY Lss and QY max reached their minimum values (0.22 and 0.81, respectively) at DAT 127–128 (end of June) contrasting with 0.54 and 0.85 (QY Lss and QY max, respectively) observed in the control group of plants (Figure 4a,b).

Both quantum yields of PSII in drought‐treated plants began to decline compared to control at DAT 70 (beginning of the May; Figure 4a,b). However, only QY max showed greater sensitivity to drought stress, differing significantly at DAT 70 (Figure 3). This result arises from the model used in Equation (1), which accounts for the provenance effect. QY Lss response to induced stress was the slowest of all five parameters tested, and its reduction in treated plants started to be significant from DAT 83 (39 days after the reduction of watering), when SRWC was 20% and reached a PWP (Figure 3 and Figure 4a,b).

At the beginning of cultivation, NPQ Lss—steady‐state non‐photochemical quenching, a protective mechanism activated when PSII can no longer utilize light energy photochemically—decreased from 2.5 (DAT 36) to 2.34 in both plant groups, until irrigation was reduced (DAT 44, early April). At DAT 70, corresponding to 26 days after the onset of irrigation reduction, stressed and control plants exhibited different responses. NPQ Lss of treated plants was slightly lower than that of the controls, and this parallel pattern continued until re‐irrigation (Figure 4c). Similar to QY max, NPQ Lss values between treated and control groups did not differ significantly until DAT 70 (Figure 3, Tables S5, S6).

Predicted SFR_R, the chlorophyll fluorescence ratio at far‐red/red used for estimating chlorophyll content, increased until the onset of irrigation reduction at DAT 44 (Figure 4d). During the stress period, the SFR_R parameter showed an unstable trend. However, the significant difference in SFR_R between control and treated plants appeared as early as DAT 57 (mid‐April), only 13 days after the onset of treatment, indicating greater sensitivity compared with the other tested parameters. Since SFR_R can serve as an estimate of chlorophyll content, the significantly higher SFR_R values observed in drought‐treated plants compared with controls at DAT 57 (Figure S3) may indicate altered chlorophyll levels under drought stress. Both SFR_R values declined until DAT 67 (late April) and then increased until DAT 88 (mid‐May), after which they stabilized for 7 days, up to DAT 95 (late May). During this period (DAT 88–95), both temperature and light intensity remained consistently high and stable. From DAT 102 (early June) onward, a decrease in SFR_R values was observed in both control and treated plants. Subsequently, SFR_R values of control plants increased slowly until the end of our experiment (from DAT 102 to 158, June, and July), due to the continued development of new shoot terminals. DAT 116 (mid‐June) was the first time from the start of the experiment when the SFR_R values for control plants exceeded drought‐treated plants, whose SFR_R values increased more slowly than the SFR_R values of control plants. Approaching severe drought stress (from DAT 102 to 123, June), SFR_R values for control plants increased from 1.3 to 1.5, and treated plants remained stable around 1.3.

The differences between needle temperature and ambient temperature $\left(∆T\right)$, reflecting plant cooling and transpiration capacity, showed a consistent trend for both control and treated plants at the beginning of the experiment (Figure 4e). Although the control and treated groups differed significantly in ∆T (Table S7), the measured values suggest that this difference is not of biological significance and is rather the result of large sample size. As SRWC reached maximum drought (soil moisture below the PWP on DAT 83 mid‐May), water in the pots became unavailable to the plants. Consequently, the difference between needle and ambient temperature sharply rose to 0.8°C for control and 1.4°C for treated plants, respectively, showing more effective transpiration cooling of control plants. During the period when the treated plants did not have available water (from DAT 83 to DAT 128), the temperature difference between the needle and ambient temperature for treated plants reached 2°C, while control plants maintained a ∆T of 0°C at the end of this drought period (Figure 4e). The increase of the temperature difference, especially in treated plants, during the water stress period was amplified by hot weather, impacting the greenhouse at the end of June (Figure S2).

### 3.4 Response of Needle Functional Traits to Recovery During the Season

After rewatering at DAT 128, both PSII quantum yields started to recover. However, the significant differences in QY Lss between control and treated plants were detectable up to DAT 147 (late July) (19 days after rewatering). In QY max, no difference between control and treated plants was observed from DAT 154 (end of July); thus, it was day 26 of the recovery period (Figure 3, Tables S4, S5).

When irrigation was restored to the treated plants, at DAT 128, NPQ Lss values for the treated plants briefly rose up to the value of 3 in DAT 132 (early July), coinciding with the peak summer temperature (Figure S2). Subsequently, NPQ Lss of the treated plants gradually decreased along with the temperature during the restoration period, converging toward the values observed in the control plants. On DAT 141 (mid‐July), as temperature, light intensity, and air humidity decreased, both groups exhibited NPQ Lss values around 2.7 (Figure 4c). NPQ Lss values maintained a significant difference between the treated and control plant groups from DAT 70 to DAT 147. At the 19th day after rewatering, NPQ Lss values were not significantly different between treated and control plant groups (Figure 3, Table S6).

After rewatering (DAT 128, late June), SFR_R values for treated plants began to gradually increase from DAT 137 (early July) toward those of control plants at the end of the experiment, and both groups reached fluorescence values in DAT 158 (late July) around 1.6 (Figure 4d). This observed increase in chlorophyll content estimation could be attributed again to the maturation of new shoots. Despite the rewatering process, the SFR_R values of stressed seedlings did not completely return to control levels, and statistically significant differences persisted throughout the rewatering period (Figure 3, Table S7). This persistence indicates a negative impact on chlorophyll content, confirming the effects of reduced watering on treated plants.

During the recovery phase, ∆T values for both treated and control plants increased to values up to 2.2°C for control plants and up to 3.1°C for treated plants in DAT 133 (early July). The sudden increase in ∆T observed in both groups can probably be attributed to daily temperature fluctuations with maxima exceeding 35°C. In addition, night temperatures in early July were also higher compared to other periods throughout the experiment (Figure S2). The high temperatures paired with low relative humidity may have led to exceeding seedlings´ transpiration cooling capacity of control group even under good water availability. The impaired cooling could also result from the reduction of transpiration area due to needle shedding in control plants. A similar trend in temperature differences between treated and control plants was observed during recovery from DAT 133 to DAT 140 (early June), with higher values in both groups compared to the period of severe stress in DAT 126 (late June). On the DAT 143 (mid‐July), ∆T decreased from 2.3°C to 0.6°C for treated plants and from 1.5°C to −0.3°C for control plants, compared to DAT 140 (Figure 4e). This variation may be attributed to the decline in temperature and relative humidity. ∆T maintained a significant difference between control and treated plants until the end of our measurements (Figure 3, Table S8).

### 3.5 Response of Needle Functional Traits to Drought and Recovery in Locally Adapted Provenances During the Season

During our 166‐day experiment, in which Scots pine seedlings were exposed to drought, differences in their responses of five key needle functional traits, QY Lss, QY max, NPQ Lss, SFR_R, and ∆T, were tested by mixed linear model (Equation 1) among the three locally adapted provenances (Tables S4–S8).

Significant differences in QY max, SFR_R, and ∆T among three provenances were observed in the initial stages, from the start of treatment (DAT 44, early April) until the soil moisture level reached the permanent wilting point (DAT 83, mid‐May), when the maximum drought was achieved. However, the effect of the provenances was not consistent for QY Lss and NPQ Lss at the initial stage of irrigation reduction.

During the period of severe drought stress when soil reached PWP and when potted water was unavailable for treated plants between DAT 83 and DAT 127 (SRWC below 20%), our analysis revealed consistent significant differences among locally adapted provenances in four of the five needle functional traits (QY Lss, QY max, SFR_R, and ∆T). However, NPQ Lss showed no significant differences between provenances during the dry season (Table S6).

After rewatering on DAT 128, the provenances continued to show differences in all needles functional traits we measured. During the subsequent recovery phase, differences in measured traits differed among the studied provenances. QY Lss showed provenance differences early in the recovery process, but these differences disappeared toward the end of the experiment (Table S4). In contrast, SFR_R showed provenance‐level differences consistently throughout the recovery period (Table S7). QY max, NPQ Lss, and ∆T showed inconsistent differentiation between provenances during this recovery phase (Tables S5, S6, S8). Thus, the differences among provenances are in this phase smaller than within provenances.

### 3.6 Heritability Estimates of Needle Functional Traits

**FIGURE 5:**
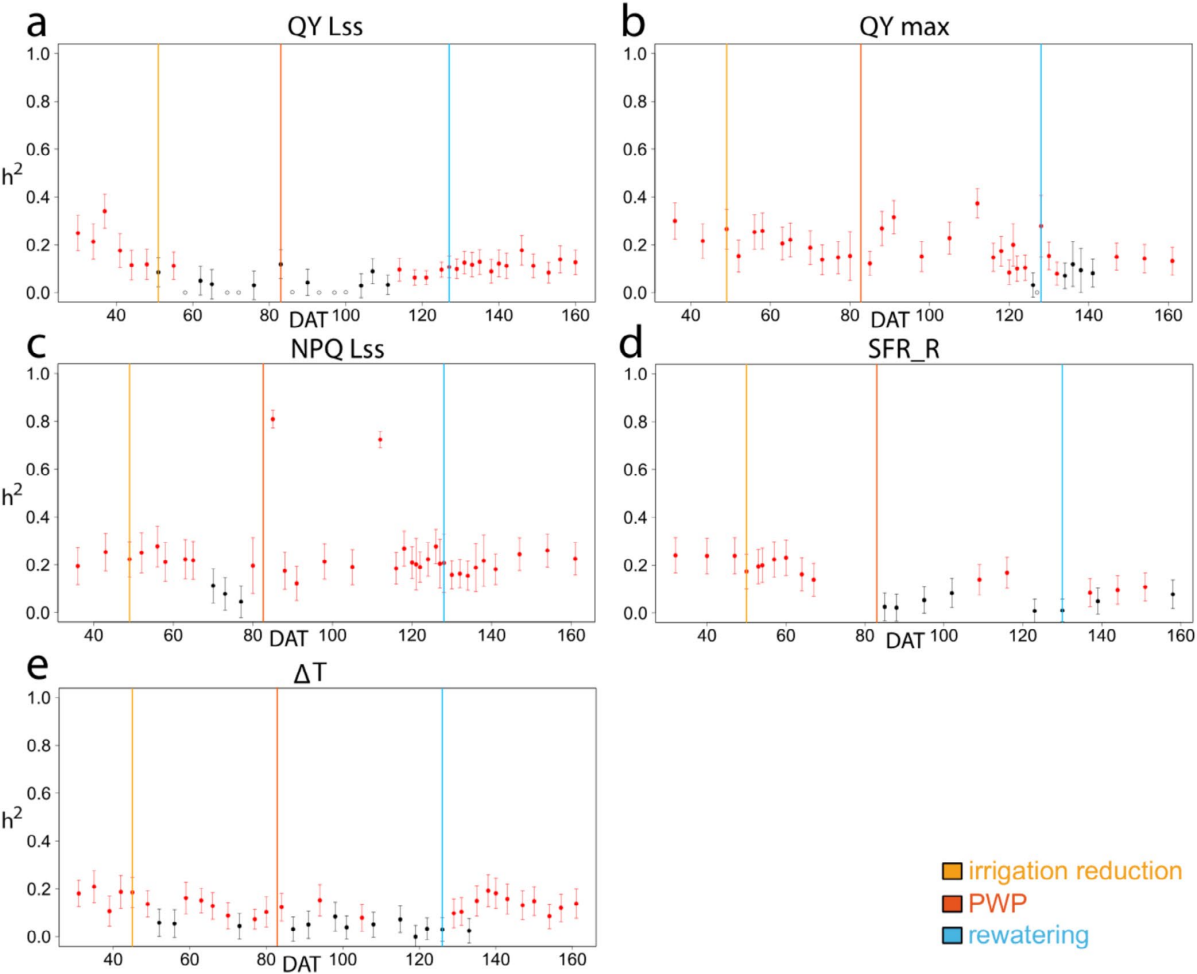
Narrow‐sense heritability (*h* ^2^, *Y*‐axis) and corresponding standard errors for five needle functional traits (a = QY Lss, b = QY max, c = NPQ Lss, d = SFR_R, e = ∆T) across the *X*‐axis: Days after transplantation (DAT 0–166). Red dots represent statistically significant, black dots represent statistically insignificant *h* ^2^ values based on LRT comparing the model with and without the random genetic term. Open dots represent zero values of *h* ^2^. The yellow line represents irrigation reduction at DAT 44, the orange line represents the permanent wilting point from DAT 83, when water was not available for the plants in this type of soil (SRWC under 20%), and the blue line represents rewatering at DAT 128.

In our study, we utilized the overall relationship (G matrix) as a parameter refining the linear mixed model (see Materials and Methods section). The genetic variability measured in single time point for growth‐related functional traits (Table S3) and functional needle traits, measured at multiple times throughout the experiment, is quantified by *h* ^*2*^ (Equation 2, Figure 5).

Consistent values of *h* ^*2*^ were observed in all measured functional traits throughout the experiments' time span. The *h* ^*2*^ values for all traits ranged from 0.06 to 0.37 (except for two extremes in NPQ Lss as described next). Given the overlapping of most confidence intervals, conducting a biological inference of the observed *h* ^*2*^ pattern becomes challenging.

Throughout the experiment, the *h* ^*2*^ of the QY max and QY Lss exhibited the highest variability, with ranges of 0–0.37 and 0–0.34, respectively. The NPQ Lss displayed a narrower range of *h* ^*2*^, from 0.05 to 0.28, excluding the two outliers recorded at DAT 85 and 112. In contrast, SFR_R and ∆T showed relatively lower variability in *h* ^*2*^ from 0 to 0.24 and from 0 to 0.21, respectively. Despite the general trends, extreme values of *h* ^*2*^ are reported at specific time points for both QY Lss and NPQ Lss traits. Notably, *h* ^*2*^ for the QY Lss was recorded as 0 at DAT 58, 69, 72, 86, 93, 97, and 100, as shown in Figure 5a. Furthermore, the LRT, used to compare the fit between the null model and the model based on Equation (1), showed a deviation from the usual trend in LRT *P*‐values for the QY Lss trait. The LRT *P*‐values were close to 0 and tended to increase as *h* ^*2*^ decreased. Assuming zero *h* ^*2*^ values in QY Lss, LRT *P*‐values reached 1 (Table S4). In addition, the NPQ Lss demonstrated distinctly high *h* ^*2*^ values of 0.81 and 0.72 at DAT 85 and 112, respectively (Figure 5c). Despite the LRT *P*‐values being 0 at these time points, the *z*‐ratios of the models, which signify the importance of random factors, were notably higher—by an order of magnitude—than the *z*‐ratios observed for NPQ Lss at other time points (Table S6).

Statistically significant *h* ^*2*^ values, as determined by the LRT (red points in Figure 5), were consistently observed in all traits up to the treatment's start date (DAT 44). Subsequently, a divergence in significance was noted, with some traits maintaining significant *h* ^*2*^ values while others did not. Specifically, the QY Lss trait exhibited non‐significant *h* ^*2*^ values at nearly all points throughout the water reduction period. Following rewatering (starting at DAT 128), the same trait's *h* ^*2*^ became significant and persisted until the end of the experiment (Figure 5a). The *h* ^*2*^ of QY max and NPQ Lss traits remained statistically significant during the stress period and after rewatering, except for few specific dates (DAT 126, 134, 136, 138, 141 and DAT 70, 73, 77) for QY max and NPQ Lss, respectively (Figure 5b,c). The significance of *h* ^*2*^ for SFR_R and ∆T traits exhibited variability both during and after the stress period. It is noteworthy that *h* ^*2*^ of ∆T predominantly remained mostly significant after rewatering (Figure 5d,e). Comprehensive results (*h* ^*2*^, standard error, *P*‐values associated with fixed factors, z‐ratio associated with random factors, LRT score) for the entire duration of the experiment, together with overall *h* ^*2*^ estimates obtained from the independent measurement analyses, are provided in Tables S3–S8.

### 3.7 Genetic Variation

The genetic structure among provenances was assessed using discriminant analysis of principal components (DAPC). The DAPC scatterplot (Figure S1) shows distinct clustering of individuals from Decin (red), Plasy (blue), and Trebon (green), with some overlap between groups. The 95% inertia ellipses illustrate the within‐group variability, and density plots along the first two discriminant functions highlight differentiation patterns among provenances. The eigenvalues indicate that the first discriminant function captures the largest proportion of genetic variance, while PCA eigenvalues suggest that only a limited number of principal components contribute meaningfully to the observed genetic differentiation.

Overall, while the genetic structure among provenances is significant, the proportion of total genetic variation that it explains is shallow (Supporting Information S1). There is no significant correlation between geographical distance and genetic differentiation, as indicated by the low correlation coefficients.

## 4 Discussion

### 4.1 Impact of Drought on Growth‐Related Functional Traits of Scots Pine Seedlings

Our study revealed a notable drought effect on all measured growth‐related functional traits, which all imply from water availability, namely, reduction in parameters NL, RCD, and EWT together with an increase in LMA. Similarly, Taeger et al. (2015) also observed a significant impact of drought on Scots pine seedlings in similar growth‐related functional traits, including a reduction in stem diameter, stem length increment, total seedling biomass, bud quantity, and increased root–stem biomass ratio and specific leaf area (inverse of LMA). It was previously recorded that smaller pines exhibited enhanced resistance, characterized by the capacity of the individual to remain unchanged during the disturbance, and greater resilience, indicating their ability to recover pre‐disturbance structures and functions, than larger sized pines (Merlin et al. 2015). Thus, some drought‐related trait reduction does not necessarily have to be negative for future seedling survival. Growth reduction following the drought event may be viewed as an acclimation strategy, enhancing overall functionality and hydraulic safety (Gessler et al. 2020). In our experiment, we observed excessive needle shedding at the end of the drought period (DAT 127), after the severest combination of stressors (high temperature combined with low relative humidity; see Figure S2), particularly in the control group seedlings. That indicates that, under extreme complex stress conditions, the adaptive capacity of pine seedlings with isohydric strategy can lead to increased drought resilience for seedlings already experienced stress conditions (Seidel and Menzel 2016). This is probably connected with already activated mechanisms for protection against oxidative stress, common to all interacting stressors, in the drought‐treated group (Noctor et al. 2014).

We concluded that this may indicate that control seedlings, in contrast to drought‐pretreated ones, were not acclimated to severe environmental stress conditions, which appeared around DAT 110–127, when a complex of interacting stressors occurred in the semi‐controlled greenhouse: increasing temperature combined with low air relative humidity (Figure S2). It also implied differences in the stress‐adaptive capacity of seedlings to a severe complex stress, depending on progeny origin: lowland progenies (P and T) exhibited lower physiological acclimation and adopted the ultimate strategy of needle shedding to reduce leaf area, whereas the montane progeny (D) exhibited only minor needle shedding, particularly in drought‐acclimated seedlings.

### 4.2 Impact of Drought on Optically Derived Needle Functional Traits

Our plants are likely to experience an amplified response to drought, especially under elevated mid‐summer temperatures. While Rehschuh et al. (2022) demonstrated the resistance of well‐watered Scots pine seedlings to elevated temperatures, parallel research by the same team (Rehschuh and Ruehr 2022) showed that drought and heat stress act synergistically to reduce light‐adapted quantum yield, net assimilation rate, and leaf cooling in pine seedlings.

In recent decade, several controlled experiments on Scots pine seedlings have been conducted, and the impact of drought on needle functional traits has been described (Nadal‐Sala et al. 2021; Rehschuh et al. 2022; Rehschuh and Ruehr 2022; Seidel et al. 2019, 2016; Seidel and Menzel 2016; Taeger et al. 2015).

The severe drought stress experienced by our samples likely triggered a notable compensatory response in quantum yield in contrast to the findings of Seidel et al. (2019). Whereas we observed a restoration of both steady‐state and maximum quantum yield of PSII, Seidel et al. (2019) documented a lack of recovery in the quantum efficiency of PSII following rewatering after spring and summer drought within the single growing season. However, recovery depends on the scale and complexity of the function examined (Gessler et al. 2020). Seidel's observations did not show a severe drop in quantum yield values during the drought; thus, the recovery was not as remarkable as in our case. Interestingly, Seidel et al. (2019) reported the compensatory response of PSII quantum yield in Scots pine seedlings exposed to a second growing season of drought stress after rewatering in a vegetation hall in contrast to greenhouse setting. Similar to our results, Rehschuh and Ruehr (2022) also observed recovery in light‐adapted quantum yield and assimilation and hydraulic traits, even after Scots pine seedlings were exposed to combined drought and heat stress in individual tree chambers. Our study also observed a comparable compensatory response in the parameter NPQ Lss immediately after rewatering following drought stress (Figure 4c).

Chlorophyll content (estimated via SFR_R) exhibited a parallel trend in both well‐watered and stressed plants during the summer period (Figure 4d), which we primarily attribute to the phenological changes. The decrease of SFR_R in both groups observed in DAT 50–70 and DAT 90–100 could be explained by the emergence of new shoots with low chlorophyll content in the newly developing needles (Gielen et al. 2000), which may result in the lower whole seedling canopy SFR_R signal. The delayed drop of SFR_R and apparently lower chlorophyll content in drought‐treated plants probably resulted from the slower development of new shoots due to water shortage. Nevertheless, in control plants, values of SFR_R continuously grew since DAT 100 corresponding to needle maturation and accumulation of chlorophylls. Contrastingly, further SFR_R reduction in the drought‐treated seedlings was followed by the similar SFR_R increase only after rewatering, which we explain by the delayed needle maturation caused by drought.

Leaf temperature is widely regarded as a reliable indicator of plant water status, stomatal conductance, and the transpiration rate (Jackson 1982; Mertens et al. 2023). The irrigation reduction combined with gradually increasing day temperatures from April to June (Figure S2) was the major cause of increased ∆T in treated seedlings. The impaired cooling of drought‐treated seedlings probably resulted from downregulated transpiration by stomatal closure as reported across 11 broadleaf evergreen woody plants (Marchin et al. 2020). Our observations are consistent with Seidel et al. (2016), reporting significant 6‐week drought‐induced reduction of stomatal conductance in Scots pine seedlings and increase in crop water stress index (calculated as the ratio of normalized leaf surface temperatures and surface temperatures of wet and dry plants). Our results point to Scots pine's conservative water use strategy based on the strong stomatal conductance regulation. In addition to transpiration, the sensible heat flux contributes to leaf cooling in conifers (Muller et al. 2021). Reduced needle length in drought‐treated seedlings could impede needle cooling by heat convection. Although we did not intentionally apply the heat stress, the daily temperature maxima exceeded frequently 30°C since the beginning of June (DAT 100), Figure S2. The sharp increase in ∆T in both well‐watered and drought‐treated seedlings is in accordance with the rise of leaf temperatures during the controlled heat wave Scots pine (Rehschuh et al. 2022).

### 4.3 Impact of Genetic Origin on Growth‐Related and Needle Functional Traits of Scots Pine Seedlings

We did not observe a significant effect of provenance on drought‐induced mortality in Scots pine seedling. This contrasts with previous findings by Seidel and Menzel (2016), who reported a significant impact of provenance. We attribute the very low seedling mortality (13 individuals) in the present study to the deliberate selection of plants that had already survived their first season overwintering under common garden conditions—individuals that had likely undergone natural preselection for better fitness, as reported in our recent study using the same locally adapted provenances (Stejskal et al. 2023).

In our study, we found no significant effect of seedling's origin within the group of control and treated plants for growth‐related parameters: RCD, LMA, and EWT. In contrast to our findings, some studies concurred on the significant effects of the provenance factor on seedling's growth (Seidel et al. 2019; Taeger et al. 2015). Variation in growth‐related parameters such as seedling height, stem diameter, and needle growth in seedlings of Scots pine was observed across different provenances (Seidel et al. 2019). The only exception, where the original provenance effect was significant, was needle length—and even then, only in control plants (Figure 2). This difference may reflect the variation in needle growth among the provenances reported by Seidel et al. (2019). Our differing conclusions regarding the provenance effect may result from the broader geographical range of provenances across Europe in the studies by Taeger et al. (2015) and Seidel et al. (2016), compared with our focus on three provenances within the Czech Republic. Despite our provenances being locally adapted as lowland or montane, with distances from their areas of origin not exceeding 300 km.

At the end of the experiment (DAT 135 and 168), following the regeneration period, we measured growth‐related functional traits (NL, RCD, LMA, and EWT). By this stage, seedlings may have undergone the compensatory growth process described by Seidel et al. (2019), in which growth rates can increase after drought stress, occasionally even exceeding typical levels. Such recovery responses could have influenced the way individual provenances reacted to the irrigation reduction.

In our investigation, we found no clear evidence that the interaction between provenance and treatment affects the ΔT during either the stress or recovery periods. This contrasts with Seidel et al. (2016), who concluded that Scots pine seedlings from various provenances exhibit distinct responses to moderate drought stress and more consistent reactions to severe drought stress. However, this interacting effect became apparent consecutively in three measurements spanning DAT 73–80, as outlined in detail in Table S8.

In our study, we observed a significant effect of provenance on most studied physiological traits, including SFR_R in all measuring days. This generally supports the conclusion by Seidel et al. (2016) that drought sensitivity and resilience in Scots pine depend on native provenances. Additionally, the significance of the provenance effect on QY max and ΔT was mostly stable during the whole experiment.

### 4.4 SNP Genotyping: Applications, Challenges, and Filtration Strategies

The integration of molecular markers into phenotypic prediction models improves accuracy and reduces bias, while replacing pedigree‐based matrices with genomic relationship matrices further refines genetic variance estimates, accelerates breeding cycles, and increases genetic gains and adaptability (Cappa et al. 2019; El‐Kassaby et al. 2024). Due to the decreasing cost of sequencing, the use of methods providing large genome‐wide SNP datasets is increasing, such as genotyping by sequencing (Elshire et al. 2011) and Rad‐Seq (Andrews et al. 2016). As these methods have limitations and require additional laboratory steps, the platforms of SNP genotyping arrays (LaFramboise 2009) are more straightforward to process with standardized laboratory and bioinformatic procedures (Pavan et al. 2020) and also provide efficient and straightforward tools leading to increased reproducibility across various research studies (Ganal et al. 2012).

The Axiom 50 K Scots pine genotyping array (PiSy50k) used in the present study was developed by Kastally et al. (2022). Although the reference genome of Scots pine is not currently available, the authors annotated the array based on the genome of the related *Pinus taeda* (Neale et al. 2014). Since Scots pine is the subject of intense research, there have been recently published an alternative Axiom 50K Scots pine SNP chip Psyl50K (Estravis Barcala et al. 2024).

Paralogs in non‐model organisms or species with incompletely analyzed reference genomes present significant challenges for SNP detection and genotyping. This issue is particularly relevant for plants, which are prone to gene duplication events (Mastretta‐Yanes et al. 2014). Paralogy‐related misalignments can lead to erroneous identification of loci as heterozygous (Verdu et al. 2016).

There is no consensus on the best practices for SNP filtering; however, several studies have demonstrated the use of heterozygosity filters (Dallaire et al. 2023; Mastretta‐Yanes et al. 2014; Verdu et al. 2016). E.g., Suissa et al. (2024) established a maximum heterozygosity threshold of 0.5 for SNP filtering in glycine species.

### 4.5 Genetic Variability of Studied Traits

The narrow‐sense heritability (*h* ^*2*^) specifically assesses the proportion of phenotypic variance attributed to additive genetic effects. Consistent *h* ^*2*^ values for all growth and for all needle functional traits throughout the experiment, ranging from 0.06 to 0.37 (excluding the extreme values discussed next), offer substantial evidence of significant additive genetic variance in the respective traits.

As reported by Ismael et al. (2022), low *h* ^*2*^ values in *Pinus radiata* were linked to drought stress conditions, primarily due to increased environmental variance. Similarly, Ehdaie and Waines (1994) documented a similar pattern in both broad‐sense and narrow‐sense *h* ^*2*^ values in wheat ( *Triticum aestivum* L.).

In the study by Ismael et al. (2022) the *h* ^*2*^ for maximum quantum yield of PSII was observed to approach zero after 5 months of water stress, primarily due to a limited number of observations. However, after 10 months of stress, the *h* ^*2*^ value for the same trait was 0.06. In contrast, within our study involving a sample size of 810 individuals, we are reporting *h* ^*2*^ values ranging from 0 to 0.37. We attribute the higher *h* ^*2*^ values in our findings to the controlled experimental conditions, which effectively reduced residual variance, conducted at a finer time scale. While comparing *h* ^*2*^ directly across plant species presents challenges, Ismael et al. (2022) concluded in their study that the imposition of moderate to severe water stress can indeed lead to lower *h* ^*2*^ values, a trend that is supported by our findings.

In the context of *h* ^*2*^ estimation, the calculation depends on the genetic relatedness among the seedlings. The genetic covariance of studied traits results from the product of genetic coancestry (relatedness) and additive genetic variance. Traditionally, *h* ^*2*^ estimates have been obtained through controlled crosses of parental individuals, using optimized half‐ and full‐sibling experimental designs (Falconer and Mackay 1996).

The Scots pine seedlings in our current study, however, originated from natural random mating (panmixia) among parents within the seed orchard. This process typically involves open pollination, with pollen grains randomly allocated onto female strobili. Given this complex pedigree structure, the so‐called animal genetic model (Henderson 1953) emerges as the preferred method for *h* ^*2*^ estimation. This model effectively utilizes all available information (genetic covariance in complex pedigreed provenances). The animal model employs either a genetic matrix based on identity‐by‐descent probabilities (ABLUP) or the actual genomic‐based relationships (as implemented here, GBLUP) as a random factor. This approach enhances the accuracy of genetic relationships among individuals, factoring both the Mendelian sampling term and historical relationships. This approach is advantageous for clarifying connections within the studied provenance, as highlighted by Gamal El‐Dien et al. (2016).

When comparing the *h* ^*2*^ values of all measured traits in our study to those obtained using the so‐called family model, which accounts for the random effect of maternal genotype (results not presented here), we consistently observed lower *h* ^*2*^ estimates. This result aligns with findings from Gamal El‐Dien et al. (2016) and Ismael et al. (2022), who also reported lower *h* ^*2*^ estimates based on GBLUP than on ABLUP models. In the study by Ismael et al. (2022), conducted research wherein the *h* ^*2*^ values for basal diameter were estimated during the drought stress period, ranging from 0.07 to 0.17 based on both ABLUP and GBLUP. In our study, we measured RCD after the recovery period, making it most comparable to the basal diameter after 6 months of water stress (BD6) reported by Ismael et al. (2022). Using the GBLUP linear model, we estimated an *h* ^2^ value of 0.23 for RCD (Table S3), whereas Ismael et al. (2022) reported a lower *h* ^2^ of 0.07 for BD6 based on the same model.

In general, estimating *h* ^*2*^ in controlled experiments poses methodological challenges when compared to *h* ^*2*^ values for the same trait evaluated in experiments conducted under natural conditions. This is because allelic effects depend on environmental conditions, and environmental variance is typically greatly reduced in controlled experimental conditions.

Čepl et al. (2016) estimated *h* ^*2*^ for the maximum quantum yield of primary PSII in situ and found a corresponding *h* ^*2*^ value of 0.04. In contrast, our estimation of *h* ^*2*^ of QY max ranged from 0.08 to 0.37. Our methodology differs from recent studies (Gil‐Muñoz et al. 2023; Wu et al. 2023), where *h* ^*2*^ was estimated separately for control and treated seedlings. In contrast, we estimated *h* ^*2*^ by pooling control and treated plants, which allowed us to leverage a larger sample size. Differences between treatments were incorporated as fixed effects in our models.

By conducting our experiment in controlled greenhouse conditions with systematic randomization of pot arrangements, we successfully minimized environmental variability (residual variance), resulting in accurate *h* ^*2*^ estimates. What distinguishes this study is its comprehensive evaluation of hundreds of pedigreed offspring under controlled conditions in a high‐throughput phenotyping facility. This stands in stark contrast to conventional forestry field experiments (Jankowski et al. 2019), which frequently contend with substantial environmental heterogeneity, known to negatively impact *h* ^*2*^ estimation (Wu et al. 2023).

### 4.6 Current Approaches to Enhancing Climate Resilience in Human‐Managed Forests

An increased tree mortality rate is predominantly driven by episodes of severe drought events, exemplified by notable instances such as the one in 2018 in the Western part of Central Europe (Buras et al. 2020). Diversifying and modifying species composition in man‐managed forests is a way to make them more resilient to future climate (IPCC 2022). Understanding species‐specific drought responses is crucial for elucidating acclimation and adaptation mechanisms. Recent research on white spruce ( *Picea glauca* ) identifies key genetic responses to drought, highlighting changes in photosynthesis, water transport, and stress signaling, with potential applications for forest resilience (Ribeyre et al. 2025). The capacity of young trees to extend into unfavorable conditions serves as a limiting factor for changes in species composition in temperate forests (Duque et al. 2015). On the one hand, seedlings from provenances of distant origin may face challenges associated with the length of the growing season and mean annual temperatures in provenance trials, as concluded by Seidel et al. (2019). Moreover, non‐native provenances could thrive under changing climate (Hazarika et al. 2021). However, in our study, the geographical gradient of studied provenances was sufficiently narrow; the influence of geography on phenology may be neglected in our study.

Drought resilience, one of the quantitative traits (Tuberosa 2012), is focusing on forest tree breeding (Ahmad et al. 2025; Gil‐Muñoz et al. 2023; Matallana‐Ramirez et al. 2021) in ongoing climate change causing more frequent periods of drought events (Samaniego et al. 2018; Satoh et al. 2022), which often shifts the climatic parameters of the forest habitat toward less favorable conditions (Seidl et al. 2014). In response, efforts are being made to integrate research findings into practical strategies for mitigating drought effects, including aligning genotypes with suitable environments, improving seedling resilience through nursery treatments, and employing landscape‐scale monitoring and predictive tools to inform management decisions (Groover et al. 2025). Thus, Scots pine serves as a viable candidate for investigating physiological plasticity under severe drought conditions and its adaptive response to drought stress (Nadal‐Sala et al. 2021; Rehschuh et al. 2022; Rehschuh and Ruehr 2022; Seidel et al. 2019; Seidel and Menzel 2016; Semerci et al. 2017; Taeger et al. 2015).

### 4.7 Limitations

Drought and heat stress often occur simultaneously in natural environments, making it challenging to separate their individual effects on plant performance. However, Rehschuh et al. (2022) demonstrated that under well‐watered conditions, Scots pine seedlings exhibited only a minimal physiological stress even when exposed to daytime temperatures exceeding 43°C. Due to constraints in statistical testing power and the additional number of replications essential for a singular treatment in genomic studies, an additional heat treatment was deemed impractical due to facility limitations, despite its relevance in simulating climatic effects, as pursued in this study.

In our study, complex stress conditions occurred naturally in a semi‐controlled greenhouse experiment in the mid‐summer (DAT 110–127; Figure S2); control seedlings exhibited excessive needle shedding compared to drought‐treated seedlings, which apparently showed the advantage of oxidative stress acclimation. Thus, it points to the importance of gradual stress hardening to oxidative stress, which leads to better resilience to naturally occurring stress conditions. Moreover, needle shedding differed markedly between the lowland progenies (Plasy and Trebon) and the montane progeny (Decin), with lowland progenies exhibiting roughly two‐thirds more needle shedding than the montane progeny. However, we assessed needle shedding on only 1 day (DAT 128, June 30) due to limitations in human resources. Thus, recording needle shedding could be a potentially sensitive functional trait to identify differences among progenies if measured on some more dense time frame.

One of the crucial responses to drought stress is a stomatal adjustment, followed by a reduction of the transpiring area, manifested on morphological level through needle shedding (Nadal‐Sala et al. 2021). The precise quantification of the reduction in leaf area requires further analyses, particularly through top‐side view RGB imaging. However, this quantification may pose challenges, especially for Scots pine seedlings due to low needle projection area. Alternatively, the quantification of dropped needles has not been accomplished during the experiment.

Our optical measurements of functional leaf traits always included the signal from the whole plant. It is essential to recognize that results obtained during the early phenophase may be affected by the formation and development of new needles. This is particularly relevant for the side view sensors averaging data from the previous and the current season. The observed effect is most noticeable for SFR_R, where fluctuations are more pronounced. At the same time, other parameters such as QY Lss, QY max, NPQ, and ∆T show a more consistent response for previous and current needle age classes. It is apparent that the attention has to be paid to efficient segmentation of needles formed in different years in phenotyping imaging since they constitute physiologically different entities with different interpretation of optical data obtained.

## 5 Conclusions

Scots pine seedlings of all provenances survived successfully over a month of absolute water scarcity, simulating expected spring drought spells in Central Europe under projected climate change scenarios. This supports the general high resilience of the species to severe drought. Also, we were able to detect considerable genetic variability observed the studied provenances across most of the assessed traits. Following more than a month of acclimation in semi‐controlled greenhouse conditions after transplanting in pots, the seedlings underwent an additional month of reduced watering until the soil moisture reached the permanent wilting point, a condition sustained for over a month, followed by a subsequent 1‐month recovery phase. Immediate responses to reduced watering were evident in all optically detected needle functional traits, including the performance of primary photosynthesis (QY Lss, QY max), photoprotective mechanism (NPQ Lss), transpiration cooling (∆T) and chlorophyll content (SFR_R). Upon resuming watering after 45 days of water unavailability, there was a gradual recovery of optically detected needle functional traits. One month after irrigation resumed, all traits displayed drought stress resilience, with NPQ Lss exhibiting immediately compensatory behavior. Notably, needle growth‐related functional traits, including needle length, needle mass per area, water content, and root collar diameter, showed drought‐induced reductions in all seedlings during the recovery phase. The estimated *h* ^2^ of optically detected traits varied widely, with photosynthesis‐related traits (QY max, QY Lss) showing the highest genetic variation, underscoring their potential for early‐age phenotyping and selection of drought‐tolerant genotypes. The selection of drought‐tolerant individuals based on their optically based needle functional traits, as employed in the present study, shows good promise for seedling and young tree phenotyping screening in nurseries and this approach fits into the concept that describes (Groover et al. 2025). We believe that the present study gives background for optically based phenotyping for the identification of individuals that are highly adapted to specific target environments.

## Conflicts of Interest

The authors declare no conflicts of interest.

## Supporting information


**Table S1:** Description of the seed origin (3 distinct local populations comprising lowland and upland ecotype).
**Figure S1:** Discriminant analysis of principal components (DAPC) scatterplot with individual density plots along the first (horizontal) and second (vertical) discriminant functions.
**Table S2:** Genetic differentiation.
**Figure S2:** Environmental variables in greenhouse conditions.
**Figure S3:** Phenotypic values of needle functional traits during drought stress and recovery.
**Table S3** Comprehensive data for growth functional traits.
**Table S4:** Comprehensive data for QY Lss trait.
**Table S5:** Comprehensive data for QY max trait.
**Table S6:** Comprehensive data for NPQ Lss trait.
**Table S7:** Comprehensive data for SFR_R trait.
**Table S8:** Comprehensive data for ∆T trait.
**Table S9:** Comprehensive data of all measurements separated into four distinct periods.

## Data Availability

Description of the data we provided in Supporting Information Section 4. Experimental data are provided in the Figshare repository: https://doi.org/10.6084/m9.figshare.29474057.
