# Supplementary material for: Drought Response and Genetic Variation in Scots Pine Seedlings' Provenances: Insights From High‐Throughput Phenotyping for Climate‐Resilient Forestry
Source: Evol Appl. 2025 Oct 7;18(10):e70157. doi: 10.1111/eva.70157 (PMC12501839; doi:10.1111/eva.70157)
Supplement: Supplementary file 1 — Table S1: Description of the seed origin (3 distinct local populations comprising lowland and upland ecotype). Figure S1: Discriminant analysis of principal components (DAPC) scatterplot with individual density plots along the first (horizontal) and second (vertical) discriminant functions. Table S2: Genetic differentiation. Figure S2: Environmental variables in greenhouse conditions. Figure S3: Phenotypic values of needle functional traits during drought stress and recovery. Table S3 Comprehensive data for growth functional traits. Table S4: Comprehensive data for QY Lss trait. Table S5: Comprehensive data for QY max trait. Table S6: Comprehensive data for NPQ Lss trait. Table S7: Comprehensive data for SFR_R trait. Table S8: Comprehensive data for ∆T trait. Table S9: Comprehensive data of all measurements separated into four distinct periods. [file EVA-18-e70157-s001.pdf]

Supporting Information

1 Material

Table S1 Description of the seed origin (three distinct local populations comprising lowland and upland ecotype). Decin trees were mostly selected in an elevation of 700 m above sea level. Climatic variables for 2021 were adopted from https://www.inmeteo.cz/. The mean heights are reported with respective standard deviations.

Description	GPS coordinates	Elevation (m.a.s.l.)	Ecotype	Mean annual precipitation (mm)	Mean annual temperature (°C)	Mean annual sunshine hours (h)	Mean height of the progeny (mm)
Plasy	49.9087617°N, 13.4427394°E	385	Lowland	489	11.1	1,524.3	49.6 ± 9.6
Trebon	49.0166106°N, 14.8247583°E	430	Lowland	623	11.1	1,571.5	51.2 ± 10.4
Decin	50.8191364°N, 14.1172131°E	465	Upland	826	8.9	1,099.9	46.5 ± 10.4

These provenances were selected for seed orchard establishment based on the actual importance of Scots pine for reforestation in respective regions. The selection of this material for this specific study was based on typical ecotypic features observed in the formerly studied progeny (Čepl et al., 2018). Plus, trees originated from semi-natural stands typical for either lowland pine or upland pine ecotypes. The scions from these trees were grafted, and the grafts were planted in the seed orchards, where we collected the seeds for the establishment of this experiment.

Čepl, J., Stejskal, J., Lhotáková, Z., Holá, D., Korecký, J., Lstibůrek, M., ... & Albrechtová, J. (2018). Heritable variation in needle spectral reflectance of Scots pine (Pinus sylvestris L.) peaks in red edge. Remote Sensing of Environment, 219, 89-98.

Figure S1 Discriminant Analysis of Principal Components (DAPC) scatterplot with individual density plots along the first (horizontal) and second (vertical) discriminant functions. The analysis included 790 individuals (189 from Decin (red), 303 from Plasy (blue), and 298 from Trebon (green)). Each dot represents an individual, with colors indicating sample origins. The 95 % inertia ellipses are shown. Pairwise distances in kilometres, along with genetic distances measured by Fst and Nei's D, are also indicated.

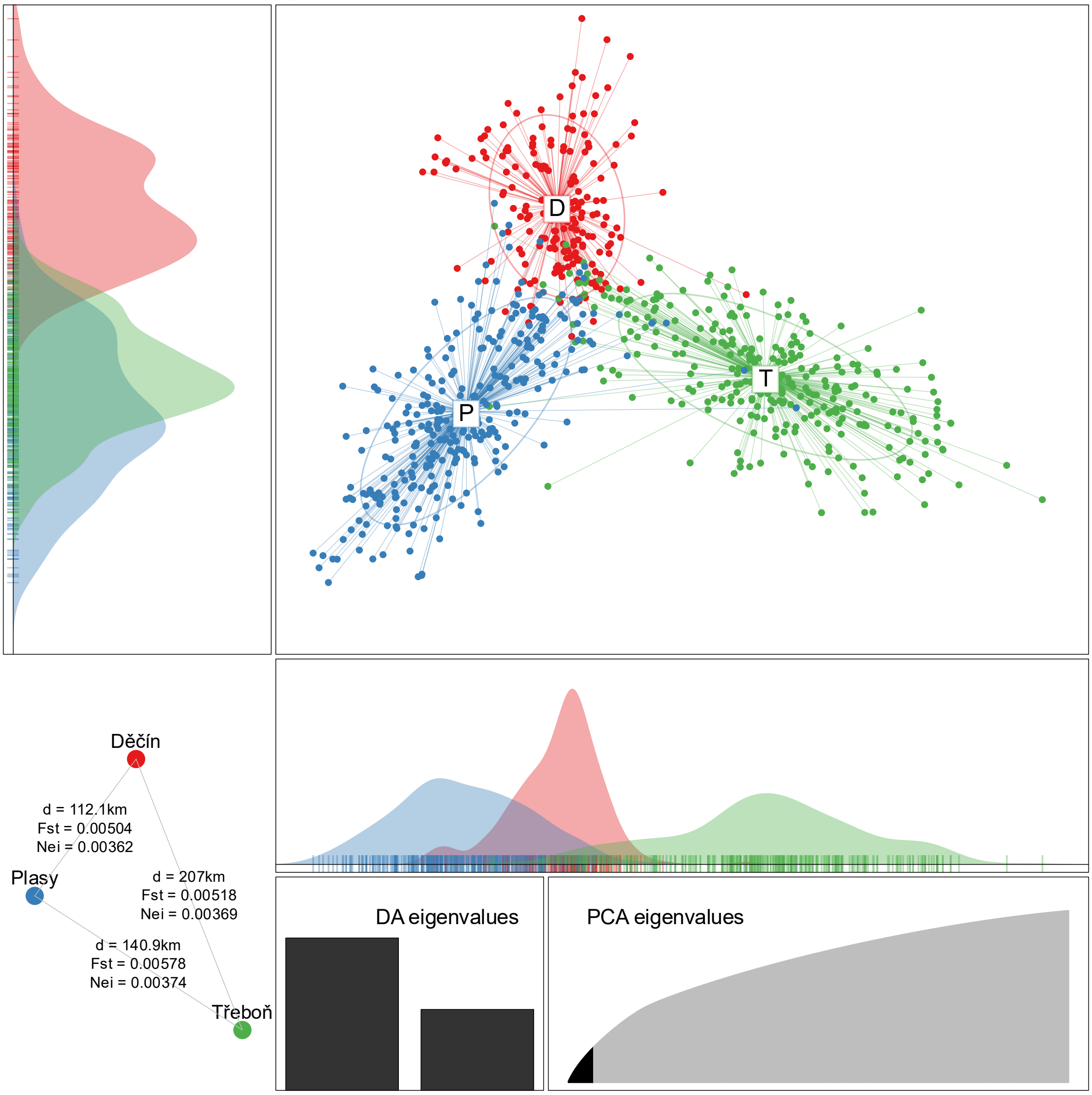

The population genetic analysis was conducted using Discriminant Analysis of Principal Components (DAPC) with functions from the R package adegenet. To balance discrimination power and overfitting, we applied the optim.a.score() function to determine the optimal number of retained Principal Components (PCs), resulting in n.pca = 40. Genetic differentiation among populations was assessed using Fst and Nei's D, as implemented in the StAMPP package. Pairwise genetic differentiation was quantified using Fst and Nei's D, with the following values:

Table S2 Genetic differentiation

Comparison	Geographical Distance (km)	Fst	Nei's D
Decin – Plasy	112.1	0.00504	0.00362
Plasy – Trebon	140.9	0.00578	0.00374
Decin – Trebon	207.0	0.00518	0.00369

2 Supporting methodology: DNA analysis-genotyping

On DAT 78 (May 11th), needles for DNA analysis were collected. From everyone, four needles were placed into the zipped bag and frozen.
Approximately 100 mg of needles per sample were cut into small pieces using a scalpel and immediately preserved in liquid nitrogen. These samples were then homogenized for three minutes at 30 Hz using an MM400 mixer mill (Retsch, Haan, Germany). Total genomic DNA was extracted using the NucleoSpin Plant II (Macherey-Nagel, Düren, Germany) following the manufacturer's protocol. DNA concentration and purity were quantified employing a NanoDrop 2000 spectrophotometer (Thermo Fisher Scientific, Madison, WI, USA), with a subset of these measurements further validated by Qubit assay (Thermo Fisher Scientific, Madison, WI, USA). The DNA's integrity was assessed on a 0.8% agarose gel. Undiluted aliquots of 45 µL DNA (mean concentration 116 ng/µL, 260/280 ratio between 1.56 and 1.93) were distributed into 96-well PCR plates. These plates were then sent packed in dry ice to SGS INSTITUT FRESENIUS GmbH, Germany, for analysis. Genotyping was conducted using the PiSy50k SNP chip Axiom array according to Kastally et al. (Kastally et al., 2022). The resulting raw data were provided in CEL file format.

Kastally, C., Niskanen, A. K., Perry, A., Kujala, S. T., Avia, K., Cervantes, S., ... & Pyhäjärvi, T. (2022). Taming the massive genome of Scots pine with PiSy50k, a new genotyping array for conifer research. The Plant Journal, 109(5), 1337-1350.

Figure S2 Environmental variables in greenhouse conditions. Y-axis: sensor readings. Lines represent a: temperature (°C), b: photosynthetic active radiation (PAR; µmol·m$^{-2, s-1}$), and c: relative air humidity (%). X-axis: days after transplantation (DAT 0-161). Grey colour shows data inside the greenhouse; the red colour shows outer conditions internal data; All day and night measurements are represented by dots. The grey and red lines connect day measurements from 7:00 AM to 6:00 PM to prevent light and temperature fluctuation during the night. Data from five sensors for inner greenhouse conditions were collected using the PlantScreen™ Modular System at PSI Research Centre, Czechia. The yellow line represents irrigation reduction at DAT 44, the orange line represents the permanent wilting point from DAT 83, when water was not available for the plants in this type of soil (SRWC under 20%), and the blue line represents rewatering at DAT 128.

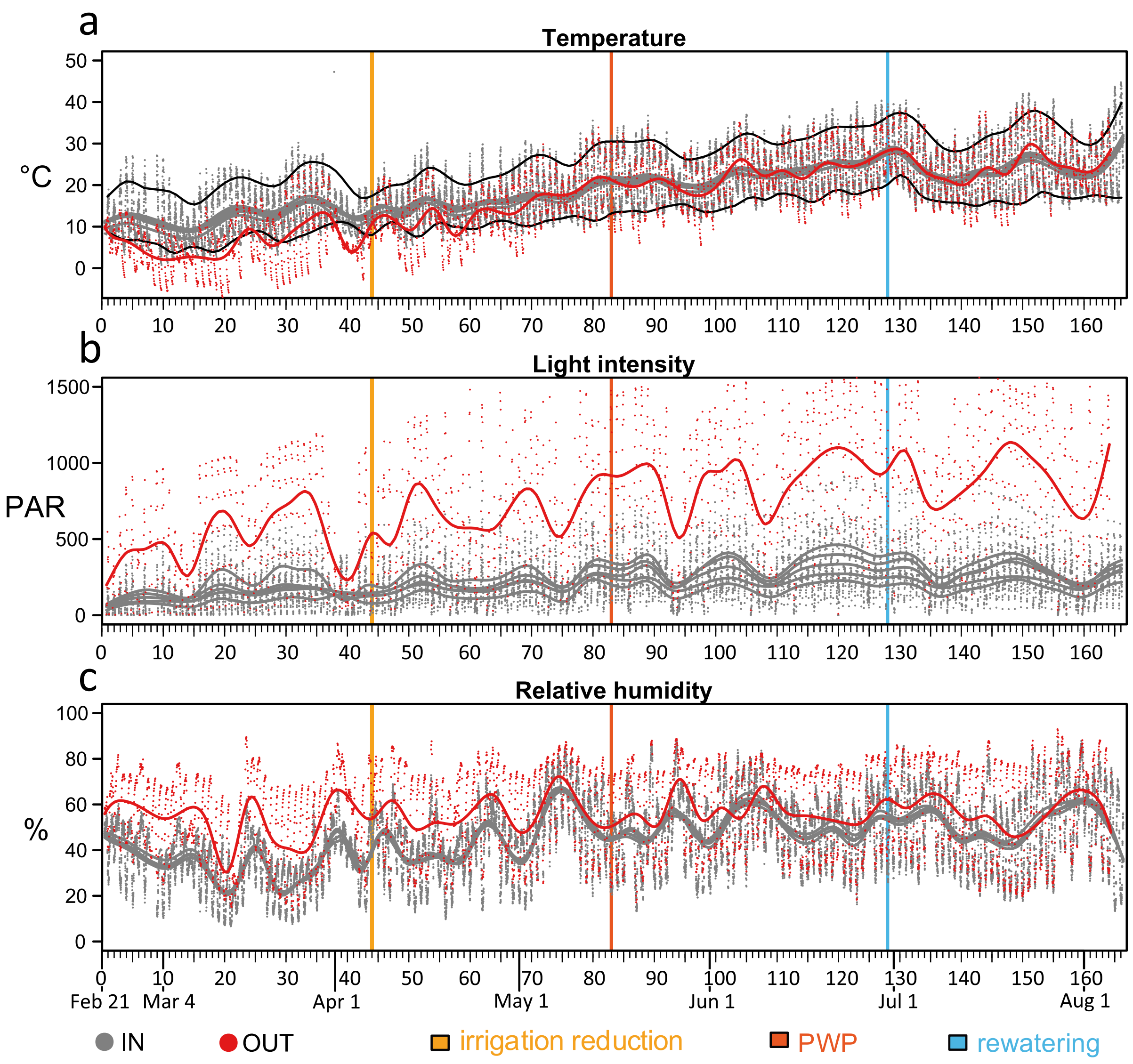

Inner temperature fluctuated between 7°C and 20°C at the experiment's onset, gradually increasing to 30°C. At 130th day after treatment (DAT, start of July), the maximum temperature of 35°C was reached, marking a peak in the seasonal temperature trend. Subsequently, a notable dip in temperature occurred at DAT 140 (around 20°C), followed by another peak at DAT 150 (35°C), (late July). Inner temperature closely reflected outer temperature variations especially in case of extreme temperatures.

Inner photosynthetic active radiation (PAR) varied from 0 to 400 µmol·m^{-2}·s^{-1} throughout the season, with notable peaks of the 400 µmol·m^{-2}·s^{-1} at DAT 120 and DAT 150 (end of June and July). A gap in PAR around 200 µmol·m^{-2}·s^{-1} (at DAT 95, 110, 135 and 160, end of May, mid-June, early July, and early August). The outer light intensity achieved two times higher than the averaged inner PAR, with higher amplitudes. Maximum PAR values were observed at DAT

120 and DAT 150 (PAR 1000 µmol·m^{-2}·s^{-1}), with corresponding declines at DAT 130 and DAT 160 (early July and early August), (PAR 600 µmol·m-2·s-1).

Both inner and outer relative air humidity exhibited comparable patterns throughout the experiment. The minimum humidity of 20% was recorded at DAT 30 (mid-March), while the maximum, around 60%, occurred at DAT 75 and DAT 160 (early May and early August).

Figure S3 Phenotypic values of needle functional traits during drought stress and recovery. Boxplots present measured values for five needle functional traits: a: QY Lss, b: QY max, c: NPQ Lss, d: SFR_R, and e: ΔT in each day after transplantation (DAT) on the X axis. The Y axis represent the trait values, with: control plants in blue boxes and treated plants in red boxes. Response of needle functional traits to drought stress and recovery in locally adapted populations during the season.

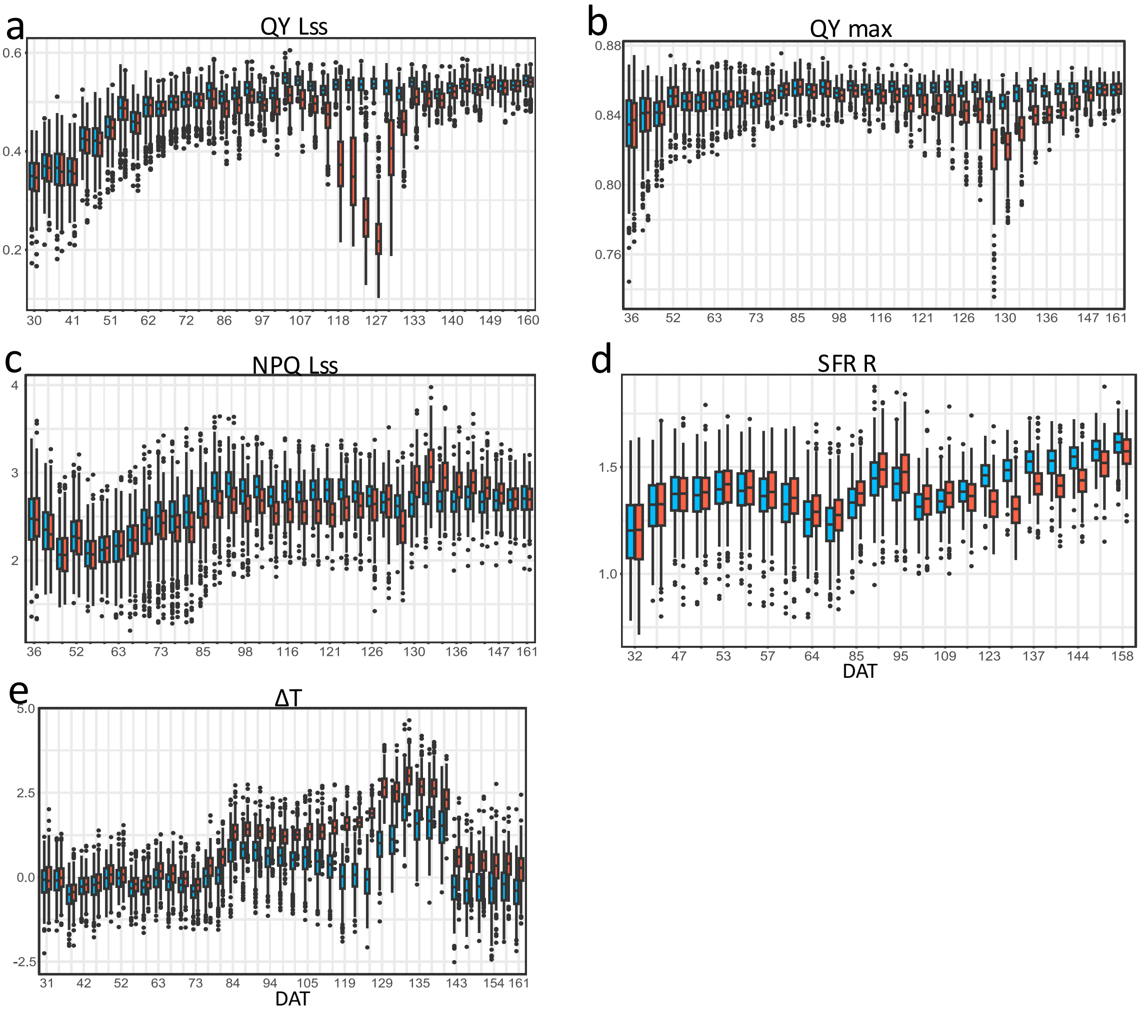

3 Supporting tables

Table S1 Comprehensive data for growth functional traits: needle length (NL), root collar diameter (RCD), leaf mass per area (LMA) and equivalent water thickness (EWT); narrow-sense heritability (h^2); standard error of estimated heritability (SE); P-values associated with fixed factors: Orchard (Orch), Treatment (TRT), and interactions between the Orchard and Treatment (Or*TRT), together with FDR adjusted Q-values (adjusted over all tests of the same DAT); z-ratio associated with random factors: Pot and G-matrix; The likelihood ratio test (LRT) P-value score compares the goodness of fit between the null model (lacking the genetic component) and the model derived from Eqn 1.

				**Orchard**		**TRT**		**Or*TRT**		**Pot**	**G-matrix**	**LRT**
	DAT	h^2	SE	*p*-val	*q*-val	*p*-val	*q*-val	*p*-val	*q*-val	z-ratio	z-rato	*P*-val
**NL**	135	0.1918	0.0728	0.0430	0.0860	<0.0001	<0.0001	0.2211	0.2653	NA	2.5498	0.0008
**RCD**	135	0.2316	0.0786	0.7072	0.7072	<0.0001	<0.0001	0.1132	0.1699	0.5489	2.8214	0.0003
**LMA**	168	0.0857	0.0607	0.1331	0.1996	<0.0001	<0.0001	0.5244	0.5244	NA	1.3982	0.0443
**EWT**	168	0.2416	0.0727	0.0001	0.0002	<0.0001	<0.0001	0.5312	0.5312	1.8881	3.1690	<0.0001

Table S2 Comprehensive data for QY Lss trait: narrow-sense heritability (h^2); standard error of estimated heritability (SE); P-values associated with fixed factors: Orchard (Orch), Treatment (TRT), and interactions between the Orchard and Treatment (Or*TRT), together with FDR adjusted Q-values (adjusted over all tests of the same DAT); z-ratio associated with random factors: Pot and G-matrix; The likelihood ratio test (LRT) P-value score compares the goodness of fit between the null model (lacking the genetic component) and the model derived from Eqn 1

**DAT**	***h²***	**SE**	**Orch**		**TRT**		**Or*TRT**		**Pot**	**G-mat**	**LRT**
			p-val	*q*-val	*p*-val	*q*-val	*p*-val	*q*-val	z-ratio	z-ratio	*P*-val
**30**	0.2939	0.0829	0.0093	0.0280	0.3319	0.4978	0.5003	0.5003	1.9733	3.3468	<0.0001
**34**	0.1969	0.0751	0.0071	0.0212	0.0691	0.1036	0.2658	0.2658	1.6301	2.5417	0.0009
**37**	0.4094	0.0801	0.0534	0.1603	0.1676	0.2514	0.3268	0.3268	2.7812	4.6160	<0.0001
**41**	0.1818	0.0734	0.4147	0.4147	0.2372	0.3746	0.2497	0.3746	0.9810	2.4093	0.0013
**44**	0.1300	0.0643	0.5120	0.5120	0.0417	0.1251	0.1519	0.2279	5.0538	1.9875	0.0097
**48**	0.1274	0.0669	0.5475	0.5475	0.0669	0.2006	0.1437	0.2156	4.7617	1.8746	0.0166
**51**	0.0810	0.0612	0.1676	0.2514	0.2822	0.2822	0.0056	0.0168	4.9985	1.3143	0.0688
**55**	0.1491	0.0640	0.6200	0.6200	0.2627	0.3941	0.1725	0.3941	5.9849	2.2827	0.0028
**58**	<0.0001	0.0579	0.3954	0.5084	0.0775	0.1949	0.0866	0.1949	3.4815	NA	0.5000
**62**	0.0363	0.0603	0.5775	0.6089	0.6089	0.6089	0.1662	0.4986	4.3489	0.6006	0.2723
**65**	0.0347	0.0609	0.1359	0.2424	0.9538	0.9538	0.1548	0.2424	3.0940	0.5684	0.2782
**69**	0.0054	0.0539	0.1274	0.3822	0.8535	0.8535	0.3920	0.5880	3.8145	0.1007	0.4580
**72**	0.0082	0.0582	0.0797	0.2392	0.6495	0.6691	0.6691	0.6691	3.9879	0.1412	0.4458
**76**	0.0403	0.0613	0.0008	0.0023	0.6376	0.7380	0.7380	0.7380	3.7825	0.6578	0.2509
**83**	0.1371	0.0646	0.0359	0.0539	<0.0001	<0.0001	0.3156	0.3156	7.3022	2.1145	0.0148
**86**	0.0107	0.0545	<0.0001	<0.0001	<0.0001	<0.0001	0.0834	0.0834	5.6171	0.1956	0.4257
**90**	0.0741	0.0610	0.0048	0.0072	<0.0001	<0.0001	0.0264	0.0264	5.7724	1.2122	0.1001
**93**	<0.0001	0.0471	<0.0001	0.0001	<0.0001	<0.0001	0.4492	0.4492	7.2429	NA	0.5000
**97**	0.0178	0.0536	<0.0001	<0.0001	<0.0001	<0.0001	0.0939	0.0939	4.6942	0.3316	0.3622
**100**	0.0328	0.0517	0.0031	0.0047	<0.0001	<0.0001	0.0112	0.0112	7.2049	0.6347	0.2709
**104**	0.0482	0.0556	0.0444	0.0666	<0.0001	<0.0001	0.5620	0.5620	6.9554	0.8653	0.1970
**107**	0.1407	0.0624	0.0054	0.0082	<0.0001	<0.0001	0.0766	0.0766	6.8528	2.2255	0.0047
**111**	0.0447	0.0454	0.0013	0.0013	<0.0001	<0.0001	0.0002	0.0002	8.0687	0.9840	0.1464
**114**	0.1141	0.0518	<0.0001	<0.0001	<0.0001	<0.0001	<0.0001	<0.0001	7.8312	2.1909	0.0029
**118**	0.0668	0.0353	<0.0001	<0.0001	<0.0001	<0.0001	<0.0001	<0.0001	9.3733	1.8952	0.0076
**121**	0.0579	0.0303	<0.0001	<0.0001	<0.0001	<0.0001	<0.0001	<0.0001	9.8283	1.9198	0.0080
**125**	0.1271	0.0389	<0.0001	<0.0001	<0.0001	<0.0001	0.0001	0.0001	9.4692	3.2845	<0.0001
**127**	0.0943	0.0442	<0.0001	0.0001	<0.0001	<0.0001	0.0001	0.0003	8.8827	2.1383	0.0048
**129**	0.0953	0.0416	<0.0001	<0.0001	<0.0001	<0.0001	<0.0001	<0.0001	8.9770	2.2975	0.0019
**131**	0.1229	0.0477	<0.0001	<0.0001	<0.0001	<0.0001	<0.0001	<0.0001	8.0976	2.5640	0.0003
**133**	0.1279	0.0543	0.0001	0.0001	<0.0001	<0.0001	0.0008	0.0009	7.2865	2.3272	0.0014
**135**	0.1770	0.0600	0.0037	0.0075	<0.0001	<0.0001	0.0103	0.0154	7.6673	2.9161	0.0002
**138**	0.1138	0.0545	0.1288	0.1931	<0.0001	<0.0001	0.1109	0.1902	7.6854	2.0800	0.0071
**140**	0.1744	0.0649	0.6623	0.6623	<0.0001	<0.0001	0.3958	0.4750	6.5491	2.6416	0.0008
**142**	0.1325	0.0573	0.1355	0.2033	0.0065	0.0194	0.2079	0.2079	7.3642	2.2853	0.0033
**146**	0.1722	0.0612	0.8411	0.8411	0.0232	0.0695	0.1591	0.2387	6.7686	2.7686	0.0002
**149**	0.1529	0.0569	0.3042	0.3893	0.3893	0.3893	0.0557	0.1672	8.1295	2.6682	0.0009
**153**	0.0933	0.0445	0.3006	0.3006	0.0133	0.0245	0.0163	0.0245	9.0396	2.0935	0.0088
**156**	0.1447	0.0599	0.2534	0.2534	0.0852	0.1277	0.0562	0.1277	7.3432	2.3944	0.0024
**160**	0.1335	0.0537	0.4673	0.4673	0.0042	0.0126	0.2860	0.4291	7.9401	2.4749	0.0014

Table S3 Comprehensive data for QY max trait: narrow-sense heritability (h^2); standard error of estimated heritability (SE); P-values associated with fixed factors: Orchard (Orch), Treatment (TRT), and interactions between the Orchard and Treatment (Or*TRT) together with FDR adjusted Q-values (adjusted over all tests of the same DAT; z-ratio associated with random factors: Pot and G-matrix; The likelihood ratio test (LRT) P-value score compares the goodness of fit between the null model (lacking the genetic component) and the model derived from Eqn 1

**DAT**	***h^2***	**SE**	**Orch**		**TRT**		**Or*TRT**		**Pot**	**G-mat**	**LRT**
			p-val	*q*-val	*p*-val	*q*-val	*p*-val	*q*-val	z-ratio	z-ratio	*P*-val
**36**	0.3265	0.0815	0.3535	0.7292	0.8588	0.9548	0.9296	0.9548	0.0657	3.7229	<0.0001
**43**	0.2289	0.0768	0.9039	0.9752	0.7524	0.9752	0.9752	0.9752	0.0260	2.8634	0.0003
**49**	0.2143	0.0806	0.4901	0.5513	0.3955	0.5085	0.3512	0.5085	0.8742	2.5711	0.0020
**52**	0.1386	0.0669	0.0108	0.0325	0.5910	0.6648	0.0971	0.1748	1.0029	2.0371	0.0063
**56**	0.2414	0.0747	0.0002	0.0005	0.9922	0.9922	0.0204	0.0459	2.1700	3.0965	<0.0001
**58**	0.2586	0.0778	<0.0001	<0.0001	0.2220	0.3330	0.0288	0.1298	0.0106	3.1648	<0.0001
**63**	0.2545	0.0756	0.0002	0.0010	0.0439	0.0987	0.0998	0.1744	1.5340	3.2106	<0.0001
**65**	0.2307	0.0736	<0.0001	<0.0001	0.0711	0.2424	0.1616	0.2424	1.7931	3.0087	<0.0001
**70**	0.1979	0.0733	0.0002	0.0007	0.0126	0.0227	0.2873	0.3232	1.8587	2.6186	0.0004
**73**	0.2322	0.0748	0.0001	0.0002	0.0111	0.0167	0.1046	0.1177	3.3436	2.9871	0.0001
**77**	0.1687	0.0710	0.0832	0.1069	<0.0001	0.0001	0.1638	0.1843	0.6549	2.3175	0.0022
**80**	0.1487	0.1026	0.0007	0.0016	0.0101	0.0140	0.2591	0.2915	2.8964	1.4298	0.0470
**85**	0.4873	0.1463	<0.0001	<0.0001	0.1497	0.2246	0.6041	0.6041	1.7304	3.0174	<0.0001
**88**	0.2913	0.0767	<0.0001	<0.0001	0.0703	0.1266	0.2210	0.3316	3.8945	3.5893	<0.0001
**91**	0.2974	0.0726	<0.0001	<0.0001	0.0234	0.0420	0.2132	0.3199	6.7702	3.9415	<0.0001
**98**	0.1927	0.0705	<0.0001	0.0001	0.0003	0.0006	0.0641	0.0961	3.2763	2.6581	0.0003
**105**	0.3189	0.0799	<0.0001	<0.0001	<0.0001	<0.0001	0.0003	0.0004	3.1317	3.7369	<0.0001
**112**	0.2848	0.0776	<0.0001	<0.0001	<0.0001	<0.0001	0.1116	0.1486	5.3750	3.5086	<0.0001
**116**	0.2251	0.0706	<0.0001	<0.0001	<0.0001	<0.0001	0.1441	0.1853	3.8092	3.0757	<0.0001
**118**	0.1719	0.0630	<0.0001	<0.0001	<0.0001	<0.0001	<0.0001	0.0001	5.8489	2.6683	0.0002
**120**	0.1017	0.0564	<0.0001	<0.0001	<0.0001	<0.0001	<0.0001	<0.0001	6.0531	1.7899	0.0127
**121**	0.2822	0.1140	<0.0001	<0.0001	<0.0001	<0.0001	0.0006	0.0007	4.8427	2.4060	0.0010
**122**	0.1330	0.0607	<0.0001	<0.0001	<0.0001	<0.0001	<0.0001	<0.0001	6.0307	2.1673	0.0035
**124**	0.1104	0.0566	<0.0001	<0.0001	<0.0001	<0.0001	0.0001	0.0001	6.5976	1.9405	0.0085
**126**	0.0872	0.0602	0.0002	0.0003	<0.0001	<0.0001	<0.0001	<0.0001	6.1464	1.4378	0.0559
**127**	<0.0001	<0.0001	0.1611	0.1812	<0.0001	<0.0001	0.0066	0.0099	3.4163	NA	0.4974
**128**	0.2438	0.1267	0.0001	0.0002	<0.0001	<0.0001	0.0028	0.0033	3.9791	1.8683	0.0166
**130**	0.1818	0.0648	0.0631	0.0710	<0.0001	<0.0001	0.0049	0.0088	6.7485	2.7638	0.0004
**132**	0.0926	0.0520	0.0276	0.0331	<0.0001	<0.0001	0.0018	0.0036	7.4961	1.7796	0.0174
**134**	0.1045	0.0610	0.0004	0.0008	<0.0001	<0.0001	0.0276	0.0331	6.1075	1.7036	0.0275
**136**	0.1294	0.0981	0.1972	0.2957	<0.0001	<0.0001	0.6527	0.7833	3.8032	1.3082	0.0679
**138**	0.0885	0.0945	0.0139	0.0335	<0.0001	<0.0001	0.5819	0.6819	4.3536	0.9330	0.1599
**141**	0.1271	0.0665	0.0412	0.0823	<0.0001	<0.0001	0.7298	0.7298	3.4916	1.8874	0.0120
**147**	0.1711	0.0616	0.4630	0.5952	<0.0001	<0.0001	0.8468	0.8468	7.0835	2.7433	0.0003
**154**	0.1521	0.0628	0.0141	0.0422	0.0680	0.1530	0.3637	0.4676	6.7833	2.3855	0.0018
**161**	0.1957	0.0684	0.0012	0.0054	0.2991	0.3845	0.2973	0.3845	5.8693	2.7919	0.0002

Table S4 Comprehensive data for NPQ Lss trait: narrow-sense heritability (h^2); standard error of estimated heritability (SE); P-values associated with fixed factors: Orchard (Orch), Treatment (TRT), and interactions between the Orchard and Treatment (Or*TRT) **together with FDR adjusted Q-values (adjusted over all tests of the same DAT**; z-ratio associated with random factors: Pot and G-matrix; The likelihood ratio test (LRT) P-value score compares the goodness of fit between the null model (lacking the genetic component) and the model derived from Eqn 1

**DAT**	***h²***	**SE**	**Orch**		**TRT**		**Or*TRT**		**Pot**	**G-mat**	**LRT**
			p-val	*q*-val	*p*-val	*q*-val	*p*-val	*q*-val	z-ratio	z-ratio	*P*-val
**36**	0.1855	0.0782	0.2502	0.7292	0.9548	0.9548	0.3646	0.7292	1.9359	2.3091	0.0046
**43**	0.2606	0.0828	0.0183	0.1096	0.3914	0.8970	0.4485	0.8970	1.7711	2.9995	0.0002
**49**	0.2772	0.0811	0.0002	0.0008	0.8064	0.8064	0.3101	0.5085	2.9426	3.2468	0.0001
**52**	0.2122	0.0832	0.0351	0.0791	0.3638	0.5458	0.5617	0.6648	2.1468	2.4686	0.0047
**56**	0.2136	0.0835	0.2430	0.4374	0.9274	0.9922	0.5069	0.7603	3.2784	2.4837	0.0056
**58**	0.1894	0.0816	0.1684	0.3031	0.7086	0.7627	0.7627	0.7627	2.0341	2.2598	0.0077
**63**	0.1773	0.0804	0.3890	0.4376	0.3851	0.4376	0.6399	0.6399	1.6280	2.1522	0.0110
**65**	0.2204	0.0829	0.1472	0.2424	0.7125	0.8069	0.7172	0.8069	1.7438	2.5653	0.0027
**70**	0.1112	0.0737	0.0002	0.0007	0.0373	0.0560	0.9620	0.9620	0.6737	1.4926	0.0603
**73**	0.1224	0.0751	0.0027	0.0049	0.0014	0.0031	0.9849	0.9849	0.6303	1.6080	0.0485
**77**	0.0557	0.0686	0.0041	0.0061	<0.0001	<0.0001	0.9049	0.9049	0.7260	0.8100	0.2118
**80**	0.2119	0.1219	0.0027	0.0048	<0.0001	<0.0001	0.8816	0.8816	0.8374	1.6937	0.0288
**85**	0.1856	0.1274	0.3718	0.4462	<0.0001	<0.0001	0.1175	0.2246	NA	1.4291	0.0440
**88**	0.1567	0.0776	0.3698	0.4161	<0.0001	<0.0001	0.9432	0.9432	0.8929	1.9800	0.0166
**91**	0.1165	0.0724	0.5165	0.5811	<0.0001	<0.0001	0.8187	0.8187	2.3614	1.5922	0.0433
**98**	0.2063	0.0759	0.5260	0.6553	<0.0001	<0.0001	0.5825	0.6553	3.5231	2.6348	0.0009
**105**	0.1710	0.0732	0.6816	0.7668	<0.0001	<0.0001	0.0549	0.0705	3.3082	2.2808	0.0038
**112**	0.2199	0.0784	0.6001	0.6001	<0.0001	<0.0001	0.1238	0.1486	4.5853	2.7084	0.0006
**116**	0.2411	0.0749	0.1687	0.1897	<0.0001	<0.0001	0.3135	0.3135	4.3908	3.0827	<0.0001
**118**	0.2619	0.0761	0.6538	0.6538	<0.0001	<0.0001	0.3442	0.3873	4.8122	3.2748	<0.0001
**120**	0.2123	0.0704	0.4807	0.4807	<0.0001	<0.0001	0.0986	0.1183	5.2138	2.9089	0.0001
**121**	0.3782	0.1405	0.8989	0.8989	<0.0001	<0.0001	0.6491	0.7303	1.9098	2.5162	0.0006
**122**	0.2459	0.0730	0.7947	0.7947	<0.0001	<0.0001	0.0396	0.0509	5.4154	3.2263	<0.0001
**124**	0.2180	0.0714	0.3210	0.3210	<0.0001	<0.0001	0.0006	0.0007	4.9314	2.9516	0.0001
**126**	0.3229	0.0804	0.0092	0.0104	<0.0001	<0.0001	<0.0001	<0.0001	4.0220	3.7461	<0.0001
**127**	0.2271	0.1123	0.0399	0.0513	0.7158	0.7158	0.0010	0.0018	0.9723	1.9587	0.0068
**128**	0.2169	0.1285	0.0350	0.0350	<0.0001	<0.0001	<0.0001	<0.0001	3.0879	1.6494	0.0247
**130**	0.1676	0.0630	0.2618	0.2618	<0.0001	<0.0001	0.0208	0.0268	5.6830	2.5964	0.0003
**132**	0.1852	0.0650	0.1738	0.1738	<0.0001	<0.0001	0.0060	0.0091	5.7390	2.7743	0.0002
**134**	0.1688	0.0657	0.6956	0.6956	<0.0001	<0.0001	0.0114	0.0170	5.7022	2.5080	0.0012
**136**	0.1713	0.1021	0.9743	0.9743	<0.0001	<0.0001	0.0732	0.1463	4.5904	1.6488	0.0312
**138**	0.2161	0.1104	0.6819	0.6819	<0.0001	<0.0001	0.3274	0.4366	4.1668	1.9088	0.0155
**141**	0.1987	0.0698	0.0856	0.1284	<0.0001	<0.0001	0.6966	0.7298	5.5611	2.7629	0.0004
**147**	0.2596	0.0737	0.0149	0.0268	<0.0001	<0.0001	0.6707	0.7546	5.3800	3.3654	<0.0001
**154**	0.2679	0.0742	0.0072	0.0325	0.4254	0.4786	0.9775	0.9775	5.4361	3.4301	<0.0001
**161**	0.2406	0.0708	0.0228	0.0685	0.4118	0.4118	0.3607	0.4058	5.7862	3.2624	<0.0001

Table S5 Comprehensive data for SFR_R trait: narrow-sense heritability (h^2); standard error of estimated heritability (SE); P-values associated with fixed factors: Orchard (Orch), Treatment (TRT), and interactions between the Orchard and Treatment (Or*TRT), together with FDR adjusted Q-values (adjusted over all tests of the same DAT); z-ratio associated with random factors: Pot and G-matrix; The likelihood ratio test (LRT) P-value score compares the goodness of fit between the null model (lacking the genetic component) and the model derived from Eqn 1

**DAT**	h^2	**SE**	**Orch**		**TRT**		**Or*TRT**		**Pot**	**G-mat**	**LRT**
			p-val	*q*-val	*p*-val	*q*-val	*p*-val	*q*-val	z-ratio	z-ratio	*P*-val
**32**	0.2399	0.0764	0.0001	0.0003	0.7652	0.7652	0.5706	0.7652	0.3780	3.0048	0.0001
**40**	0.2261	0.0756	<0.0001	<0.0001	0.8196	0.8261	0.8261	0.8261	NA	2.8754	0.0002
**47**	0.2126	0.0754	<0.0001	<0.0001	0.7748	0.7748	0.3194	0.4792	NA	2.7232	0.0004
**50**	0.1421	0.0709	<0.0001	<0.0001	0.2654	0.2820	0.2820	0.2820	NA	1.9690	0.0108
**53**	0.1684	0.0706	<0.0001	<0.0001	0.0842	0.1263	0.2069	0.2069	NA	2.3282	0.0019
**54**	0.1834	0.0715	<0.0001	<0.0001	0.0653	0.0979	0.1211	0.1211	NA	2.4948	0.0008
**57**	0.2057	0.0736	<0.0001	<0.0001	0.0138	0.0207	0.0480	0.0480	NA	2.7033	0.0003
**60**	0.2162	0.0741	<0.0001	<0.0001	0.0012	0.0018	0.1480	0.1480	NA	2.8109	0.0002
**64**	0.1547	0.0708	<0.0001	<0.0001	<0.0001	<0.0001	0.6563	0.6563	NA	2.1413	0.0047
**67**	0.1275	0.0693	<0.0001	<0.0001	<0.0001	<0.0001	0.8421	0.8421	NA	1.8137	0.0162
**88**	0.1069	0.1018	<0.0001	<0.0001	0.0113	0.0255	0.3538	0.4161	0.7509	1.0406	0.1146
**95**	0.1271	0.0941	<0.0001	<0.0001	0.0002	0.0003	0.0560	0.0560	0.7098	1.3339	0.0431
**102**	0.1495	0.1024	<0.0001	<0.0001	0.0011	0.0017	0.0891	0.0891	1.1901	1.4365	0.0393
**109**	0.2250	0.1037	<0.0001	<0.0001	<0.0001	0.0001	0.0039	0.0039	1.1888	2.0975	0.0022
**116**	0.2198	0.1075	<0.0001	<0.0001	<0.0001	0.0001	0.0112	0.0168	1.6297	1.9820	0.0047
**123**	0.0781	0.0932	<0.0001	<0.0001	<0.0001	<0.0001	0.0268	0.0268	1.7299	0.8337	0.1599
**130**	0.0930	0.0914	<0.0001	<0.0001	<0.0001	<0.0001	0.0188	0.0268	2.9812	1.0107	0.1099
**137**	0.2711	0.1193	<0.0001	<0.0001	<0.0001	<0.0001	0.6880	0.6880	2.4267	2.1818	0.0029
**139**	0.1628	0.1070	<0.0001	<0.0001	<0.0001	<0.0001	0.7064	0.7064	2.2155	1.4938	0.0369
**144**	0.2131	0.1133	<0.0001	<0.0001	<0.0001	<0.0001	0.2174	0.2174	1.1858	1.8288	0.0122
**151**	0.2071	0.1071	<0.0001	<0.0001	<0.0001	<0.0001	0.1329	0.1329	1.1649	1.8798	0.0070
**158**	0.1347	0.1038	<0.0001	<0.0001	<0.0001	<0.0001	0.0924	0.0924	1.5434	1.2803	0.0625
**121**	0.3782	0.1405	0.8989	0.8989	<0.0001	<0.0001	0.6491	0.7303	1.9098	2.5162	0.0006
**122**	0.2459	0.0730	0.7947	0.7947	<0.0001	<0.0001	0.0396	0.0509	5.4154	3.2263	<0.0001
**124**	0.2180	0.0714	0.3210	0.3210	<0.0001	<0.0001	0.0006	0.0007	4.9314	2.9516	0.0001
**126**	0.3229	0.0804	0.0092	0.0104	<0.0001	<0.0001	<0.0001	<0.0001	4.0220	3.7461	<0.0001
**127**	0.2271	0.1123	0.0399	0.0513	0.7158	0.7158	0.0010	0.0018	0.9723	1.9587	0.0068
**128**	0.2169	0.1285	0.0350	0.0350	<0.0001	<0.0001	<0.0001	<0.0001	3.0879	1.6494	0.0247
**130**	0.1676	0.0630	0.2618	0.2618	<0.0001	<0.0001	0.0208	0.0268	5.6830	2.5964	0.0003
**132**	0.1852	0.0650	0.1738	0.1738	<0.0001	<0.0001	0.0060	0.0091	5.7390	2.7743	0.0002
**134**	0.1688	0.0657	0.6956	0.6956	<0.0001	<0.0001	0.0114	0.0170	5.7022	2.5080	0.0012
**136**	0.1713	0.1021	0.9743	0.9743	<0.0001	<0.0001	0.0732	0.1463	4.5904	1.6488	0.0312
**138**	0.2161	0.1104	0.6819	0.6819	<0.0001	<0.0001	0.3274	0.4366	4.1668	1.9088	0.0155
**141**	0.1987	0.0698	0.0856	0.1284	<0.0001	<0.0001	0.6966	0.7298	5.5611	2.7629	0.0004
**147**	0.2596	0.0737	0.0149	0.0268	<0.0001	<0.0001	0.6707	0.7546	5.3800	3.3654	<0.0001
**154**	0.2679	0.0742	0.0072	0.0325	0.4254	0.4786	0.9775	0.9775	5.4361	3.4301	<0.0001
**161**	0.2406	0.0708	0.0228	0.0685	0.4118	0.4118	0.3607	0.4058	5.7862	3.2624	<0.0001

Table S6 Comprehensive data for ΔT trait: narrow-sense heritability (h^2); standard error of estimated heritability (SE); P-values associated with fixed factors: Orchard (Orch), Treatment (TRT), and interactions between the Orchard and Treatment (Or*TRT), together with FDR adjusted Q-values (adjusted over all tests of the same DAT); z-ratio associated with random factors: Pot and G-matrix; The likelihood ratio test (LRT) P-value score compares the goodness of fit between the null model (lacking the genetic component) and the model derived from Eqn 1

DAT	h^2	SE	Orch		TRT		Or*TRT		Pot	G-mat	LRT
			p-val	*q*-val	*p*-val	*q*-val	*p*-val	*q*-val	z-ratio	z-ratio	*P*-val
**31**	0.4381	0.0783	<0.0001	<0.0001	0.2257	0.3386	0.9974	0.9974	7.1633	5.1543	<0.0001
**35**	0.3283	0.0797	<0.0001	<0.0001	0.0511	0.0766	0.9371	0.9371	5.7853	3.8761	<0.0001
**39**	0.2287	0.0782	<0.0001	<0.0001	0.0246	0.0369	0.2463	0.2463	4.4302	2.8190	0.0008
**42**	0.3875	0.0867	<0.0001	<0.0001	0.1128	0.1692	0.3433	0.3433	4.1166	4.0856	<0.0001
**45**	0.4010	0.0841	0.0001	0.0003	0.0115	0.0172	0.0713	0.0713	5.0263	4.3485	<0.0001
**49**	0.2476	0.0693	<0.0001	<0.0001	0.0061	0.0184	0.1528	0.3439	7.7502	3.4708	<0.0001
**52**	0.1615	0.0724	<0.0001	0.0004	0.0005	0.0021	0.9114	0.9114	5.6060	2.1873	0.0089
**56**	0.2329	0.0800	<0.0001	<0.0001	<0.0001	<0.0001	0.7918	0.9922	4.2366	2.8029	0.0008
**59**	0.3703	0.0860	0.0001	0.0001	<0.0001	<0.0001	0.3344	0.3344	4.1899	3.9556	<0.0001
**63**	0.3220	0.0685	0.0049	0.0146	<0.0001	<0.0001	0.1163	0.1744	7.5987	4.4923	<0.0001
**66**	0.2577	0.0697	0.0048	0.0072	<0.0001	<0.0001	0.4426	0.4426	7.0880	3.6065	<0.0001
**70**	0.1850	0.0705	0.0008	0.0019	<0.0001	<0.0001	0.1225	0.1575	5.9982	2.5726	0.0011
**73**	0.2193	0.0765	<0.0001	<0.0001	<0.0001	<0.0001	0.0180	0.0232	3.6209	2.7688	0.0004
**77**	0.1975	0.0613	<0.0001	<0.0001	<0.0001	<0.0001	0.0002	0.0004	7.9739	3.2148	<0.0001
**80**	0.1772	0.0728	<0.0001	<0.0001	<0.0001	<0.0001	0.0109	0.0140	3.1792	2.3740	0.0026
**84**	0.2456	0.0729	0.0006	0.0009	<0.0001	<0.0001	0.2436	0.2436	5.8315	3.2669	<0.0001
**87**	0.1072	0.0640	<0.0001	<0.0001	<0.0001	<0.0001	0.0059	0.0059	2.4401	1.6576	0.0214
**91**	0.1291	0.0707	<0.0001	0.0001	<0.0001	<0.0001	0.3981	0.5119	0.9049	1.7992	0.0197
**94**	0.2627	0.0793	0.0001	0.0002	<0.0001	<0.0001	0.4117	0.4117	2.5497	3.1650	0.0001
**98**	0.1921	0.0749	0.0001	0.0002	<0.0001	<0.0001	0.7175	0.7175	1.9695	2.4952	0.0016
**101**	0.1790	0.0698	0.0002	0.0003	<0.0001	<0.0001	0.9392	0.9392	5.0940	2.5122	0.0011
**105**	0.3021	0.0813	0.0001	0.0002	<0.0001	<0.0001	0.9680	0.9680	2.1684	3.4969	<0.0001
**108**	0.1877	0.0721	0.0006	0.0009	<0.0001	<0.0001	0.5681	0.5681	5.4039	2.5420	0.0014
**115**	0.2056	0.0742	<0.0001	<0.0001	<0.0001	<0.0001	0.1890	0.1890	3.4747	2.6851	0.0004
**119**	0.0857	0.0605	<0.0001	<0.0001	<0.0001	<0.0001	0.3983	0.3983	5.1439	1.4068	0.0549
**122**	0.1516	0.0648	0.0007	0.0011	<0.0001	<0.0001	0.4985	0.5608	6.6503	2.3045	0.0028
**126**	0.1491	0.0675	0.0009	0.0012	<0.0001	<0.0001	0.2521	0.2521	5.0301	2.1722	0.0043
**129**	0.1430	0.0673	0.2979	0.3574	<0.0001	<0.0001	0.7762	0.7762	4.6583	2.0925	0.0071
**131**	0.1882	0.0693	0.4183	0.5019	<0.0001	<0.0001	0.8379	0.8379	5.4374	2.6524	0.0007
**133**	0.0866	0.0628	0.0003	0.0004	<0.0001	<0.0001	0.4543	0.4543	5.4752	1.3724	0.0718
**135**	0.2504	0.0771	0.7278	0.7278	<0.0001	<0.0001	0.3826	0.4591	4.4443	3.1216	0.0001
**138**	0.2877	0.0795	0.6722	0.6819	<0.0001	<0.0001	0.1091	0.1902	5.1470	3.4440	<0.0001
**140**	0.3195	0.0803	0.3307	0.4750	<0.0001	<0.0001	0.1220	0.2439	4.9132	3.7441	<0.0001
**143**	0.3671	0.0847	0.0007	0.0010	<0.0001	<0.0001	0.0274	0.0274	3.0894	3.9883	<0.0001
**147**	0.2772	0.0782	0.0117	0.0264	<0.0001	<0.0001	0.1403	0.2105	4.2671	3.3671	<0.0001
**150**	0.3801	0.0836	0.0399	0.0598	<0.0001	<0.0001	0.0833	0.0833	4.0079	4.1685	<0.0001
**154**	0.2741	0.0740	0.2203	0.3305	<0.0001	<0.0001	0.1376	0.2476	6.3271	3.5536	<0.0001
**157**	0.3145	0.0802	0.2183	0.3274	<0.0001	<0.0001	0.4148	0.4148	5.0145	3.6984	<0.0001
**161**	0.3128	0.0807	0.1123	0.2526	<0.0001	<0.0001	0.1426	0.2567	3.4641	3.6381	<0.0001

Table S9: Comprehensive data of all measurements separated into four distinct periods: before treatment, during the initial treatment phase until the permanent wilting point (PWP) is reached, after reaching PWP, and during the recovery phase. Narrow-sense heritability (h^2); standard error of estimated heritability (SE); P-values associated with fixed factors: Orchard, Treatment (TRT), and interactions between the Orchard and Treatment (Or*TRT), together with FDR adjusted Q-values (adjusted over all tests of the same DAT); The extended model was an expansion of the one described in Eqn. 1. It incorporated an autoregressive correlation structure (AR1) to account for temporal autocorrelation of repeated measurements within individual plants. Additionally, random genetic effects and pot effects were estimated separately for each measurement time point; h^2 was estimated as the ratio of the averaged genetic variance to the sum of averaged genetic variance, averaged pot variance, and residual variance.

**Trait**	**Period**	***h*²**	**SE**	**Orch**		**TRT**		**Or*TRT**	
				p-val	*q*-val	*p*-val	*q*-val	*q*-val	*q*-val
**QY Lss**	Before treatment	0.2936	0.0136	0.1412	0.3025	0.0363	0.0949	0.0380	0.0949
	Treatment (until PWP)	0.2408	0.0136	0.0543	0.0814	0.0436	0.0726	0.2867	0.3584
	Treatment (after PWP)	0.1005	0.0049	0.0001	0.0002	<0.0001	<0.0001	<0.0001	<0.0001
	Recovery phase	0.1081	0.0062	0.1564	0.2347	<0.0001	<0.0001	0.0261	0.0489
**QY max**	Before treatment	0.0557	0.0091	0.4374	0.6646	0.8109	0.8109	0.6874	0.7932
	Treatment (until PWP)	0.0111	0.0010	<0.0001	<0.0001	<0.0001	<0.0001	<0.0001	<0.0001
	Treatment (after PWP)	*Model did not converged*							
	Recovery phase	0.0693	0.0051	0.0016	0.0034	<0.0001	<0.0001	0.2151	0.2933
**NPQ Lss**	Before treatment	0.0367	0.0081	0.0015	0.0075	0.5359	0.7307	0.3470	0.6506
	Treatment (until PWP)	0.0109	0.0021	0.6273	0.6721	0.0022	0.0041	0.8934	0.8934
	Treatment (after PWP)	0.0339	0.0032	0.4382	0.4382	<0.0001	<0.0001	0.0059	0.0078
	Recovery phase	0.0088	0.0014	0.0789	0.1315	<0.0001	<0.0001	0.4403	0.4403
**SFR_R**	Before treatment	0.0836	0.0089	<0.0001	<0.0001	0.7464	0.7997	0.6287	0.7859
	Treatment (until PWP)	0.0030	0.0012	<0.0001	<0.0001	0.0007	0.0015	0.4523	0.5219
	Treatment (after PWP)	0.0816	0.0092	<0.0001	<0.0001	0.0004	0.0006	0.0758	0.0910
	Recovery phase	0.0192	0.0042	<0.0001	<0.0001	<0.0001	<0.0001	0.2449	0.3061
**ΔT**	Before treatment	0.0757	0.0076	<0.0001	<0.0001	0.0047	0.0175	0.4430	0.6646
	Treatment (until PWP)	0.0937	0.0073	<0.0001	<0.0001	<0.0001	<0.0001	0.0720	0.0982
	Treatment (after PWP)	0.0754	0.0047	<0.0001	<0.0001	<0.0001	<0.0001	0.2962	0.3231
	Recovery phase	0.0388	0.0028	0.3946	0.4227	<0.0001	<0.0001	0.3541	0.4086

4 Data availability

Repository: https://doi.org/10.6084/m9.figshare.29474057
The data used in the manuscript are organized according to the:

1 Functional traits
Functional traits measured and measurement frequency are divided into two categories:
1.1 Growth-Related (e.g., NL, RCD, LMA, EWT)
1.2 Optically Assessed (e.g., SFR_R, QY Lss, QY max, NPQ Lss ΔT)
These traits are summarized in Table 1 of the manuscript.

2 Genomic data
2.1 790ind_31611SNPs
2.2 SNPs_ID for G matrix
2.3 G matrix_790ind
